# Preclinical NCI-MPACT: prospective modeling of the mutation-based NCI-MPACT clinical trial therapeutic strategy in patient-derived xenograft models

**DOI:** 10.3389/fonc.2025.1571635

**Published:** 2025-05-19

**Authors:** Yvonne A. Evrard, Sergio Y. Alcoser, Michael Mullendore, Li Chen, Chih-Jian Lih, Vishnuprabha Rahul Kannan, Vivekananda Datta, Lindsay Dutko, Shahanawaz Jiwani, Lawrence V. Rubinstein, Yingdong Zhao, P. Mickey Williams, Alida Palmisano, Laura Kuhlmann, Mel Simpson, Shivaani Kummar, Biswajit Das, Chris Karlovich, Eric Polley, Ming-Chung Li, Alice P. Chen, Melinda G. Hollingshead, James H. Doroshow

**Affiliations:** 1 Applied and Developmental Research Directorate (ADRD), Frederick National Laboratory for Cancer Research, Frederick, MD, United States; 2 Division of Cancer Treatment and Diagnosis, National Cancer Institute, Bethesda, MD, United States; 3 Molecular Characterization Laboratory, Frederick National Laboratory for Cancer Research, National Cancer Institute, Frederick, MD, United States; 4 General Dynamics Information Technology (GDIT), Falls Church, VA, United States; 5 Division of Hematology/Medical Oncology, School of Medicine, Oregon Health & Science University, Portland, OR, United States; 6 Department of Public Health Sciences, University of Chicago, Chicago, IL, United States; 7 Center for Cancer Research, National Cancer Institute, Bethesda, MD, United States

**Keywords:** precision medicine, NCI-MPACT, patient-derived models, DNA damage repair, next-generation sequencing, targeted agents, MGMT deficiency

## Abstract

**Purpose:**

The National Cancer Institute’s Molecular Profiling-Based Assignment of Cancer Therapy (NCI-MPACT) randomized phase 2 clinical trial assessed the utility of applying tumor DNA sequencing to treatment selection. Here, we report the results of a companion preclinical study in patient-derived xenograft (PDX) models to evaluate how each tumor responded to each of the treatment regimens studied in the NCI-MPACT trial instead of simply to the specific regimen targeting the study-actionable mutation of interest (aMOI).

**Methods:**

Fifty-one PDX models (46 with and 5 without NCI-MPACT aMOIs) were tested against both the arm that would have been assigned in the NCI-MPACT trial as well as every other study regimen: (1) veliparib plus temozolomide or (2) adavosertib plus carboplatin (targeting the DNA repair pathway); (3) everolimus (targeting the PI3K pathway); and (4) trametinib (targeting the RAS/RAF/MEK pathway). Durability of response was measured by relative median time to tumor quadrupling event-free survival (EFSx4 ≥ 2), and duration of tumor regression.

**Results:**

Eleven of 50 models (22%) treated with veliparib plus temozolomide responded according to one or both metrics, as did 2/47 models (4.2%) treated with adavosertib plus carboplatin, and 2/46 models (4.3%) treated with trametinib; no models responded to everolimus. Follow-up studies demonstrated that temozolomide drove the activity of the veliparib plus temozolomide combination and drug sensitivity to temozolomide correlated with MGMT deficiency.

**Conclusion:**

This prospective preclinical study confirmed the modest response rates in the NCI-MPACT clinical trial. Substantial responses to temozolomide suggest that this drug represents an effective treatment for patients with MGMT deficiency, regardless of cancer type.

## 1 Introduction

**Figure 1:**
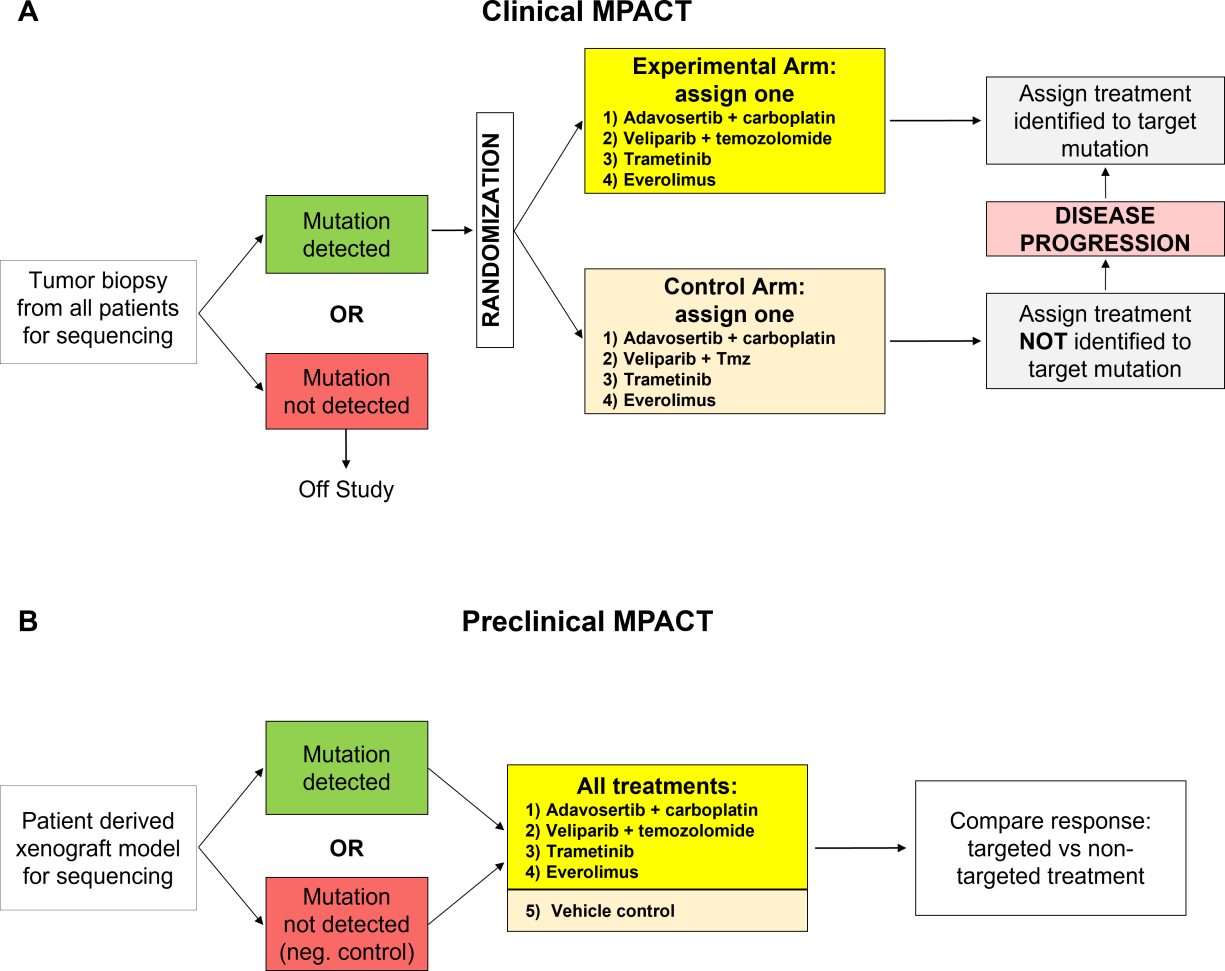
Study design for the MPACT trials. **(A)** Study design for the NCI-MPACT clinical trial (NCT01827384): Tumor biopsies collected from consenting study patients were sequenced for specific study actionable mutations of interest (aMOI); patients with such mutations were then randomized to a study regimen if additional clinical eligibility criteria were met (1). Patients randomized to a non-targeted arm were able to cross over to their targeted arm at disease progression. **(B)** Study design for the preclinical MPACT trial: Tumor specimens were collected under NCI-sponsored tissue procurement protocols and used to generate patient derived xenograft (PDX) mouse models. Tumors were sequenced for specific aMOI and mice bearing each model received all 4 NCI-MPACT treatment regiments as well as the appropriate vehicle controls. Models without an aMOI were included as additional negative controls.

The NCI Molecular Profiling-Based Assignment of Cancer Therapy (NCI-MPACT) clinical trial (NCT01827384) assessed the utility of applying tumor DNA sequencing to treatment selection by comparing the efficacy of 4 study regimens: the PARP inhibitor veliparib with the alkylating agent temozolomide, or the WEE1 tyrosine kinase inhibitor adavosertib plus the alkylating agent carboplatin (targeting the DNA repair pathway); the mTOR inhibitor everolimus (targeting the PI3K pathway); and the MEK inhibitor trametinib (targeting the RAS/RAF/MEK pathway). Patients harboring *TP53* mutations believed to be insensitive to veliparib and temozolomide were assigned to the adavosertib plus carboplatin arm. Patients with an actionable mutation of interest (aMOI) defined by the trial (**Supplementary Table S1**) were randomized either to the experimental arm and received treatment matching the aberrant genomic pathway detected in their tumor, or to the control arm and received 1 of the same 4 regimens not matched to their aMOI (**Figure 1A**). This design allowed for an unbiased comparison of targeted treatment performance with a parallel control arm rather than historical response rates. The trial reported modest activity: the objective response rate, measured as a complete or confirmed partial response (PR/CR) per Response Evaluation Criteria in Solid Tumors (RECIST) v1.1 from 49 patients in the experimental arm was 2% (95% CI: 0%, 10.9%); 1 of 20 patients (5%) in the experimental trametinib cohort had a PR. There were no responders in the other cohorts. Clinical accrual to the everolimus and veliparib plus temozolomide cohorts did not reach the 12-patient threshold for interim analysis. A confounding factor was the significantly higher pretreatment dropout rate in the control arm (22%) compared to the experimental arm (6%; *p* = 0.038), indicating that, although arm assignment was blinded, some patients may have had prior tumor mutation profiling performed, knowledge that may have influenced their decision to participate (1).

To further evaluate the efficacy of these molecularly targeted treatments, we performed a companion preclinical study in patient-derived xenograft (PDX) models selected from the National Cancer Institute’s (NCI) Patient-Derived Models Repository (PDMR) (https://pdmr.cancer.gov) to evaluate how patient-derived models would respond to each of the 4 treatment regimens rather than simply the regimen targeting the aMOI detected in the model. NCI PDMR models are quality-controlled, early-passage, and clinically annotated to be a resource for drug discovery efforts (2). Single-agent studies were also conducted to establish whether responses were driven by one drug or the combination. Furthermore, this preclinical study allowed us to identify new genomic alterations—not considered in the original clinical trial—likely to predict response to therapy in a histologically agnostic manner.

Deficiency in the expression of MGMT (O-6-Methylguanine-DNA Methyltransferase) either through low protein expression and/or silencing of the *MGMT* promoter, along with a functional DNA mismatch repair (MMR) pathway, is associated with temozolomide activity in several cancer types; in patients with glioblastoma, *MGMT* promoter methylation status is predictive of response to the drug (3–7). To understand how MGMT deficiency affects response to temozolomide in PDX models, we measured *MGMT* promoter methylation status using an analytically validated PCR-based assay and MGMT protein expression by immunohistochemistry (IHC). The mRNA expression data available for 5348 PDX tumor samples representing 1056 distinct models in the NCI PDMR were used to correlate *MGMT* mRNA levels with promoter methylation status and with response to temozolomide in this study.

Here, we describe the findings from the preclinical companion study to the NCI-MPACT clinical trial and demonstrate the value of using the well characterized NCI PDMR models to support interpretation of precision oncology studies, and of assigning treatment with temozolomide-based regimens dependent on MGMT deficiency across a spectrum of tumor histologies.

## 2 Methods

### 2.1 PDX models

A total of 51 PDX models were included in the study: 46 based on the presence of an aMOI that would have made the patient donor eligible to participate on the NCI-MPACT clinical trial (**Table 1** and **Figure 1B**) and 5 models lacking an aMOI as negative controls; diagnosis was histologically agnostic to reflect the clinical study design. The models were not derived from patients enrolled in the clinical MPACT study. The majority of the models were generated in the Biological Testing Branch, Developmental Therapeutics Program, Division of Cancer Treatment and Diagnosis (DCTD) at the Frederick National Laboratory for Cancer Research (FNLCR) from tumor specimens collected from patients at clinical centers across the United States and are available through the NCI PDMR (https://pdmr.cancer.gov). Tumor specimens were collected under NCI-sponsored tissue procurement protocols with institutional review board approval; investigators obtained written informed consent from each participant for the use of their delinked specimens to genetically characterize and generate patient-derived models and to make these models available to researchers along with limited clinical information. Several models selected were derived from the same originating patient but from different longitudinal time points or anatomic locations to explore model and lineage heterogeneity (indicated in **Table 1**).

**Table 1:** Patient-derived xenograft models tested in the preclinical NCI-MPACT study.

PDX ID	Tumor type	Clinical aMOI per NCI-MPACT	Clinical arm assignment	Response to study regimens
114551-080-T	Salivary gland carcinoma	TP53 (R175H) [0.5]	Adavosertib + Carboplatin	
245127-232-R	Lip/oral cavity squamous cell carcinoma	TP53 (R213*) [1.0]; PIK3CA (E545K) [0.59]; HRAS (G12S) [0.49]		
466636-057-R	Pancreatic adenocarcinoma	TP53 (R213*) [0.99]; KRAS (G12R) [0.5]		
692163-330-T	Uterine leiomyosarcoma	TP53 (R248Q) [1.0]		Velip+Tmz
779769-127-R	Rectal adenocarcinoma	TP53 (R273H) [1.0]; KRAS (A146T) [0.67]		
997726-040-R	Squamous lung cell carcinoma	TP53 (R273L) [0.93]; PIK3CA (E545K) [0.78]		Velip+Tmz Tmz
BL0293-F563^	Urothelial/bladder cancer	TP53 (R248Q) [1.0]		Velip + Tmz Tmz
BL0382-F1232^	Urothelial/bladder cancer	TP53 (E336*) [1.0]		Velip+Tmz
CN0375-F725†	Colon adenocarcinoma	TP53 (R175H) [1.0]; KRAS (A146T) [0.5]		
CN0446-F447†	Colon adenocarcinoma	TP53 (R273H) [1.0]		
LG0556-F006†	Lung adenocarcinoma	TP53 (R273L) [1.0]		
LG0567-F671†	Lung adenocarcinoma	TP53 (R273L) [0.9]; KRAS (G12C) [0.5]		
ST0110-F1568†	Gastrointestinal stromal tumor	TP53 (R282W) [1.0]		
LG0520-F434†	Squamous cell lung carcinoma	ERCC1 (Q67*)	Velip+Tmz	Velip+Tmz Tmz
235635-245-T	Cervix adenocarcinoma	PIK3CA (Y1021C) [0.31]	Everolimus	
261386-189-R	Urothelial/bladder cancer, NOS	PIK3CA (E545K) [0.5]		
283339-068-R	Vaginal cancer, NOS	PIK3CA (H1047R) [0.99]		
743489-274-T	Renal cell carcinoma, NOS	PIK3CA (N345K) [0.5]		
743489-281-T	Renal cell carcinoma, NOS	PIK3CA (N345K) [0.4-0.5]; TP53 (R213*) [0.1]		
BL0269-F402†	Bladder cancer	PIK3CA (H1047R) [0.53]		
128128-338-R	Melanoma	BRAF (V600K) [0.66]	Trametinib	Velip+Tmz
172845-121-B	Colon adenocarcinoma	KRAS (G12D) [0.67]; PIK3CA (E545K) [0.5]		Velip+Tmz
172845-121-T	Colon adenocarcinoma	KRAS (G12D) [0.67]; PIK3CA (E545K) [0.5]		Velip+Tmz
172845-142-T	Colon adenocarcinoma	KRAS (G12D) [0.67]; PIK3CA (E545K) [0.5]		
174941-126-T	Melanoma	BRAF (V600E) [0.75]		
248138-237-R	Hurthle cell neoplasm	NF1 (R1534*) [0.92]		
292921-168-R	Pancreatic adenocarcinoma	KRAS (G12D) [0.65]		
327498-153-R	Carcinosarcoma of the uterus	KRAS (G12C)		
349418-098-R	Lung adenocarcinoma	BRAF (V600E) [0.56]		
521955-158-R2	Pancreatic adenocarcinoma	KRAS (G12D) [0.6]		
521955-158-R3	Pancreatic adenocarcinoma	KRAS (G12D) [0.6]	Trametinib	
521955-158-R4	Pancreatic adenocarcinoma	KRAS (G12D) [0.6]		
521955-158-R6	Pancreatic adenocarcinoma	KRAS (G12D) [0.55]		
521955-158-R7	Pancreatic adenocarcinoma	KRAS (G12D) [0.6]		
563396-261-R	Melanoma	BRAF (V600E) [0.8]		
625472-104-R	Colon adenocarcinoma	BRAF (V600E) [0.98]; PIK3CA (C420R) [0.5]; MSH2 (A230Lfs*16) [0.5]		
782815-120-R	Colon adenocarcinoma	NRAS (Q61R) [0.99]		
CN0330-F216^	Colon adenocarcinoma	KRAS (G13D) [0.67]; AKT1 (E17K) [0.51]		Velip+Tmz Tmz
CN0428-F1126†	Colon adenocarcinoma	KRAS (G12A) [0.55]		
LG0481-F231*	Lung adenocarcinoma	KRAS (G12C) [0.9]; PIK3CA (E542K) [0.5-0.6]		
LG1197-F385*	Squamous cell lung carcinoma	KRAS (G12C) [0.6]		
172845-288-R	Colon adenocarcinoma	PIK3CA (E545K) [0.75]; KRAS (G12D) [0.65]	Everolimus Trametinib	Velip+Tmz
287614-091-R	Squamous cell lung carcinoma	NF1 (S749*) [0.98]; TP53 (R342*) [0.95]	Trametinib Adav+Carbo	
692585-246-R	Squamous lung cell carcinoma	TP53 (R273H) [0.43]; PIK3CA (E545K) [0.35]	Adav+Carbo Everolimus	
746718-042-R	Transitional cell carcinoma - urothelial	PIK3CA (E545K) [0.64]; NF1 (W1258*) [0.53]	Trametinib Everolimus	
997537-175-T	Colon adenocarcinoma	PTEN (K267Rfs*9) [0.5]; BRAF (V600E) [0.47]; TP53 (R273C) [0.49]	Trametinib Everolimus Adav+Carbo	
767577-098-T	Chondrosarcoma	none	none	
941425-263-T	Mesothelioma	none	none	Adav+carbo Carbo
952719-076-R	Lung adenocarcinoma	none	none	
BL0479-F1894^	Neuroendocrine carcinoma	none	none	Trametinib
SA0426-F1136^	Non-uterine leiomyosarcoma	none	none	Velip+Tmz Tmz Adav+carbo

PDMR model nomenclature for the PDX ID is comprised of a randomized patient ID, with a 3-digit collection code and letter indicating the type of tissue collected for model generation (Biopsy (T), Resection (R), CTC/Blood (B)). Within the aMOI column, asterisks within gene variant parenthesis indicate a mutation causing premature truncation of the protein, fs, a frameshift mutation. NOS, not otherwise specified. Models highlighted in yellow/orange were derived from the same originating patient from different longitudinal time points or anatomic locations. Models that met EFSx4 ≥ 2 and/or regression (PR/CR) response criteria to study agents are indicated in the right most column. **^**Models developed by The Jackson Laboratory (Bar Harbor, ME) available from the NCI Patient Derived Models Repository (NCI PDMR; Frederick, MD; https://pdmr.cancer.gov). † Models obtained from The Jackson Laboratory (Bar Harbor, ME).

A board-certified pathologist reviewed hematoxylin and eosin (H&E) stained PDX specimens from every model at passaging to confirm consistent histology with the originating patient’s diagnosis (8). Percent human genomic DNA content was also assessed from each PDX tumor by qRT-PCR (9), and short tandem repeat loci profiling (AmpFLSTR™ Identifiler™ PCR Amplification Kit, Applied Biosystems, CA, US) was completed on all models to validate model identity. Whole exome sequencing (WES) and whole transcriptome sequencing (RNASeq) were performed as described on the NCI PDMR Website and in the **Supplementary Materials**.

FNLCR is accredited by the Association for Assessment and Accreditation of Laboratory Animal Care International and follows the Public Health Service Policy for the Care and Use of Laboratory Animals. All studies were conducted according to an approved animal care and use committee protocol in accordance with procedures outlined in the “Guide for Care and Use of Laboratory Animals 8th Edition” (10).

### 2.2 Statistical considerations

A minimum of 6–10 models per drug cohort, regardless of the specific aMOI in the respective pathway of interest, were included. Targeted treatment assignment was considered based on the top aMOIs detected; for top variant allele frequencies within 15% of each other, the model was considered eligible for multiple targeted regimens. After 15 models derived from 8 patients containing KRAS mutations became available, we expanded the trametinib cohort to include tumors with additional NCI-MPACT aMOIs affecting the RAS/RAF/MEK pathway and evaluated whether the specific dominant aMOI affected response to targeted treatment. Once 46 models with at least one aMOI were included, additional PDX models were not needed for the comparison with the MPACT clinical trial. Five models lacking any NCI-MPACT aMOI served as negative controls. It should be noted that, since the pre-clinically tested PDX models did not correspond to the patients in the clinical trial, it was not possible to derive a formal comparison between the pre-clinical and clinical results by calculating a correlation coefficient. It was only possible to make an overall informal comparison of the results.

Association between *MGMT* deficiency status and response to temozolomide-based treatment in the analyzed PDX models was determined using the Fisher’s exact test.

### 2.3 PDX preclinical studies

Sex-matched NSG mice (NOD.Cg-Prkdc^scid^Il2rg^tm1Wjl^/SzJ; NCI Animal Production Program, Frederick, MD) were implanted subcutaneously with fragments from viably cryopreserved PDX tumor fragments as described in the NCI PDMR standard operating procedures (https://pdmr.cancer.gov/sops). Veliparib (ABT-888; NSC 752840), adavosertib (AZD1775; NSC 754352), trametinib (NSC 758246), everolimus (NSC 733504), temozolomide (NSC 362856), and carboplatin (NSC 241240) were obtained through the NCI Developmental Therapeutics Program (DCTD, NCI; Rockville, MD). Preclinical studies were conducted by the Biological Testing Branch (NCI-Frederick, Frederick, MD).

Mice were housed in sterile individually ventilated polycarbonate cages on RAIR HD SuperMouse 750™ ventilated racks outfitted with automatic watering and HEPA-filtered supply and exhaust air (Lab Products, Aberdeen, MD). All animals were maintained in a strict barrier facility on a 12-hour light/dark cycle and were provided sterilized food ad libitum.

Tumor-bearing mice were staged with a median tumor size of 200 mm^3^. Median passage of tumors for studies was 3 (range, passage 2–8) (**Supplementary Table S2**). Prior to drug treatment, the animals were randomized into groups using a commercial software program (Study Director, Studylog Systems, Inc.). There were no restrictions except for the adavosertib-treated animals, for whom food was withheld 2 hours before and 2 hours after dosing.

Each model was tested against all 4 of the NCI-MPACT drug regimens—adavosertib plus carboplatin, everolimus, temozolomide plus veliparib, and trametinib—with appropriate vehicle controls. A subset of models had follow-up studies comparing combination to single agent responses. Study drugs were administered to mice at doses based on the recommended phase 2 clinical doses and schedules (**Table 2**). Response was assessed using two independent metrics for depth and durability of response as defined below (11). Tumor size and body weight were measured 2-3x weekly during the study. Tumor size was monitored by bidirectional caliper measurements, and the tumor volumes (mm^3^) were calculated as (tumor length in mm × [tumor width in mm]^2^)/2 (12). Data collection was performed using the StudyLog software program StudyDirector (Studylog Systems, Inc.). For all models, the stopping point was set as either a maximal superficial tumor burden of 4000 mm^3^ or 300 days after start of treatment.

**Table 2:** Dosing regimens in the preclinical study.

Study Agent	Preclinical Dose (route of administration) (number of mice/group)	Schedule
Vehicle	12.6 g Sorbitol and 0.62 g Citric Acid Monohydrate per 120 mL Distilled Water (oral) (16)	Twice daily x 14 (7 days), repeat every 4 weeks
	10% DMSO/90% D5W(oral) (16)	Daily x 5 days every 4 weeks
Everolimus	1.94 mg/kg (oral) (6-8)	Daily x 28 days
Trametinib	0.39 mg/kg (oral) (6-8)	Daily x 28 days
Veliparib	7.75 mg/kg (oral) (6-8)	Twice daily x14 (7 days) every 4 weeks
Temozolomide	50 mg/kg (oral) (6-8)	Daily x 5 days every 4 weeks
Adavosertib	20 mg/kg (oral) (6-8)	Twice daily x 5 (2.5 days) every 3 weeks
Carboplatin	80 mg/kg (intravenous) (6-8)	Day 1, rest 20 days

Nine groups with n=16 mice for vehicle control and n=6–8 for single-agent or double-agent treatment arms administered as described. Vehicle control was used as comparator for all response calculations. D5W, dextrose 5% in water.

### 2.4 Relative median time to tumor quadrupling event free survival

The relative median time to event-free survival (EFSx4) is derived from time to tumor quadrupling or to the end of the study period relative to the vehicle control arm; this metric provides a means to quantify the durability of response to agents across different models with different dosing schedules (2, 11, 13). In these studies, a cut-off of EFSx4 ≥ 2 (2-fold delay in tumor quadrupling) in at least one experiment was used as it generally correlated with models that achieved a tumor regression. EFSx4 values were not reported for studies with substantial drug-related toxicity or that failed quality control.

### 2.5 Partial response and complete response duration

The difference in tumor volume on-study compared to staging was determined for individual tumor-bearing animals across on-treatment and off-treatment time points. Partial response (PR) was defined as the median tumor volume for the group having a ≥30% decrease in tumor volume for more than one consecutive tumor volume measurement (generally a 3–5-day separation between measurements) when at least 60% of the animals in that group were still alive. A complete response (CR) was defined as the median tumor volume for the group being ≤60 mm^3^ (limit of accurate measurement based on average thickness of the skin on the flank in control, tumor-free, NSG host animals) for more than one consecutive tumor volume measurement when at least 60% of the animals were still alive. Models that reached PR and/or CR are presented in **Supplementary Table S2**.

### 2.6 Methylation-specific PCR assay for *MGMT* promoter methylation

*MGMT* promoter methylation status was measured in tumor samples using an analytically validated, PCR-based assay developed and validated by the Molecular Characterization Laboratory at the Frederick National Laboratory for Cancer Research in Frederick, MD. Assay validation experiments reporting analytical sensitivity, specificity, and reproducibility are provided in **Supplementary Materials**. Briefly, DNA was extracted from formalin-fixed, paraffin embedded (FFPE) tissues using the Qiagen FFPE DNA/RNA Kit as described; DNA (approximately 50 ng) was converted from unmethylated cytosine residues into uracil residues using sodium bisulfite treatment (Qiagen EpiTect Bisulfite Kit). Real-time PCR methylation analysis was performed using the MethyLight assay with methylation-specific primers for sensitive discrimination occurring at the PCR amplification level (Qiagen EpiTect MethyLight PCR + ROX Vial Kit). To evaluate the relative methylation level, the percentage of methylated reference (PMR) was calculated for each sample by dividing the quantity mean of *MGMT* sample/methylated positive control by the quantity mean of *ACTB* sample/methylated positive control (Applied Biosystems QuantStudio ViiA7 software). Verification included a manual review of standard curve QC metrics, positive and negative control QC metrics, and PMR calculation by designated laboratory personnel, confirming adequate PCR efficiency and quality. For clinical applications, the limit of reporting has been established at ≥ 3% methylation with a reporting threshold of PMR ≥ 2 such that samples with results lower than a PMR of 2 are reported as negative (14) (**Supplementary Materials**).

### 2.7 MGMT immunohistochemistry

The assay was performed on FFPE-fixed tumor sections as previously described (7) using the rabbit anti-MGMT antibody clone EPR4397 (Abcam, ab108630, Boston, MA, USA). The tumor content cutoff was a minimum of 100 tumor nuclei per slide (> 1000 average for all samples). A tumor nuclear staining cutoff of ≥ 30% tumor content was set to define low MGMT expression based on the IHC scoring system for patients with glioblastoma multiforme (15). All MGMT IHC scoring was reported by a pathologist blinded to the results of the xenograft studies. For each model, non-treated PDX tumor samples from 2 passages were evaluated to assess intra-model consistency in MGMT protein expression across passages.

### 2.8 OncoPrint visualization of top genomic alterations

Top gene mutations and copy number alterations were identified for PDX models responding to veliparib plus temozolomide using cBioPortal (16, 17). Only likely-oncogenic genetic alterations detected in over 10% of the samples were plotted. The OncoPrint query considered mutations, putative copy number alterations, and mRNA up- or over-expression outside a z-score threshold of ± 2 (as determined based in RNA Seq V2 RSEM (18)).

### 2.9 Whole exome and transcriptome sequencing

Whole exome and transcriptome sequencing were performed as described in the **Supplementary Methods** across 5348 specimens derived from 1056 PDX tumors with different histologies in the PDMR.

Of the 51 tumor models selected for this study, 20 (39.2%) had confirmed oncogenic mutations in the DNA repair pathway, with the majority (17/20, 85%) being oncogenic *TP53* mutations; 18/51 (35.3%) models harbored mutations in the PI3K signaling pathway, with 16/18 (89%) oncogenic *PIK3CA* mutations; 30/51 (58.8%) models harbored mutations in the RAS/RAF/MEK signaling pathway, with 19/30 (63.3%) oncogenic *KRAS* mutations and 6/30 (20%) oncogenic *BRAF* mutations. Eighteen of 51 (35.3%) models harbored oncogenic mutations in more than one pathway, of which 5/18 (27.8%) would have been eligible for multiple NCI-MPACT treatment regimens (**Table 1** and **Supplementary Table S2**).

### 2.10 Bioinformatics data analyses for WES and RNASeq datasets

Bioinformatics data analyses for WES and RNA-Seq datasets were performed as described in the **Supplementary Methods**.

## 3 Results

### 3.1 NCI-MPACT agent activity across all PDX models

Fifty-one PDX models were derived from 21 histologies isolated from a total of 43 patients. This included 46 models with at least one NCI-MPACT aMOI (minimum 6–10 per cohort) and 5 models without NCI-MPACT aMOIs as negative controls. The most common dominant aMOIs reported were TP53 (16/46 [34.7%] models) and KRAS (14/46 [30.4%] models); subsequently the respective cohorts (targeting DNA damage response and RAS/RAF/MEK pathway respectively) were expanded to evaluate the effect of distinct aMOIs within the same pathway on targeted treatment. The most common malignancy was colon adenocarcinoma (11 PDX models derived from 8 patients), followed by pancreatic adenocarcinoma (7 models derived from 3 patients), and lung adenocarcinoma, lung squamous cell carcinoma, and urothelial/bladder cancer (5 models for each histology, each derived from an independent patient). Multiple PDX models were derived from 3 patients, using tumor material collected and implanted on different dates and from different specimen types (including resections [R], blood-derived circulating tumor cells [B], and 18-gauge tissue biopsies [T]), which allowed us to evaluate the molecular heterogeneity of tumors and models.

Eighteen out of 51 models (35.3%) harbored multiple NCI-MPACT aMOIs in 2-3 genetic pathways, and 5/51 (9.8%) models did not have any NCI-MPACT aMOIs and were included as negative controls. Each PDX model received all 4 clinical NCI-MPACT drug regimens, administered at recommended mouse-equivalent phase 2 doses, with appropriate vehicle controls; a subset of models received corresponding single agents. EFSx4 values were not reported for studies/models with substantial toxicity or that failed quality control.

**Figure 2:**
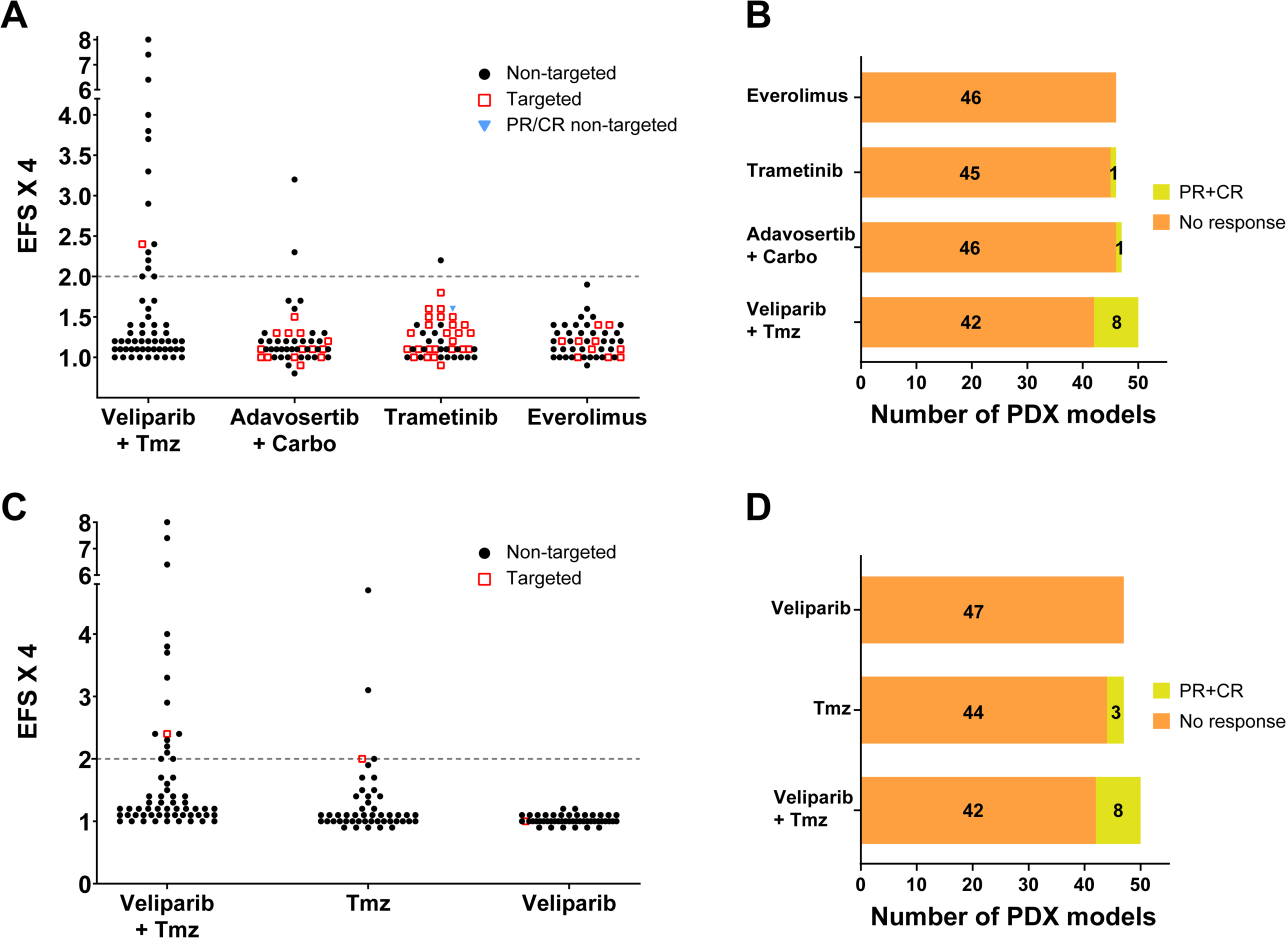
PDX responses to treatment regimens. **(A)** PDX model antitumor responses to the 4 NCI-MPACT treatment regimens based on event free survival to tumor quadrupling (EFSx4) ≥ 2. Red squares: PDX models receiving targeted treatment based on presence of NCI-MPACT clinical aMOI; black dots: PDX models receiving non-targeted treatment. Blue triangle: PDX models that achieved PR/CR receiving non-targeted treatment. **(B)** Total number of partial or complete responses (PR/CR) for all evaluable PDX models on the 4 NCI-MPACT treatment regimens. **(C)** PDX model antitumor responses to veliparib and/or temozolomide therapy based on EFSx4 ≥ 2. Red squares: PDX models harboring an NCI-MPACT clinical aMOI in the DNA repair pathway; black dots: PDX models receiving non-targeted treatment. **(D)** Total number of PR/CR in evaluable PDX models receiving veliparib and/or temozolomide treatment.

Responses to the preclinical NCI-MPACT treatment regimens measured by the quantifiable response threshold EFSx4 ≥ 2 are shown in **Figure 2A**; model responses by overall response (i.e., PR or CR) are shown in **Figure 2B**; all responding models are listed in **Table 1** and **Supplementary Table S2**. Of 13 models that achieved EFSx4 ≥ 2 on any of the 4 treatment arms in at least one experiment, 8 also met the criteria for PR/CR (≥30% regression); an additional model only met the PR regression criteria without reaching the EFSx4 ≥ 2 threshold.

Applying the threshold of EFSx4 ≥ 2, 11 of 50 (22%; 90% CI [13%, 34%]) models responded to the veliparib plus temozolomide combination in at least one experiment (**Figure 2B**). Two of these models (both derived from the same colon adenocarcinoma patient 172845) were defined as marginal responders, with a repeat experiment delivering EFSx4 values below the response threshold (**Supplementary Table S2**); model 172845-121-T was derived from a tumor biopsy of a liver metastatic lesion, whereas model 172845-288-R was derived from the resection of an adrenal mass that was unresponsive to clinical treatment. Only one responsive model (LG0520-F434, squamous cell lung carcinoma) harbored a mutation in an NCI-MPACT aMOI that would have made it eligible for this combination. Six out of the 11 responding models (54.5%) did not harbor an NCI-MPACT study aMOI in the DNA damage repair (DDR) pathway (**Supplementary Table S2**), similar to the 4/8 (50%) of models responding based on PR/CR criteria (**Supplementary Table S2**). Although the response rate was higher for the models with a DDR aMOI (31% vs 18%), because the sample sizes were small, the difference does not attain statistical significance (2-sided p-value = 0.30 by Fisher’s exact test). It should be noted that, as the preclinical PDX models were not derived from the same patients who were enrolled in the clinical trial, it was not possible to derive a formal comparison between the preclinical and clinical results by calculating a correlation coefficient; an overall informal comparison of the results could not be performed.

Two of 47 models (4.2%), neither with an NCI-MPACT aMOI, responded (EFSx4 ≥ 2) to adavosertib plus carboplatin including model SA0426-F1136 which had a confirmed PR. Two of 46 treated models (4.3%; 90% CI [1%, 12%]) responded to trametinib: one had an EFSx4 ≥ 2 and lacked an actionable NCI-MPACT mutation; the second model did not reach the EFSx4 threshold for response but did achieve a short-lasting PR to trametinib and had an actionable KRAS mutation although the allele frequency would not have made the model eligible for this treatment. None of the 46 models responded to everolimus.

### 3.2 Activity of single agent temozolomide

In follow-up studies, four of the 11 models (36%) that responded to the combination treatment of veliparib plus temozolomide also met the response threshold (EFSx4 ≥ 2) with temozolomide treatment alone (**Table 3** and **Supplementary Table S2**), and an additional model achieved a PR. No model responded to single agent veliparib by either metric (**Figures 2C, D**). These results indicate the antitumor activity of the combination was driven by temozolomide, with potentiation of temozolomide activity by veliparib in the remaining 6/11 (54.5%) models that responded only in the combination drug arm. Only 1/5 (20%) of models responding (EFSx4 and regression) to single-agent temozolomide had a confirmed NCI-MPACT aMOI that would have made it eligible for this treatment in the clinical trial. Three of the 5 models that responded (EFSx4 and regression) to temozolomide alone had confirmed PR/CRs. All 5 of the models that responded to single agent temozolomide (EFSx4 and regression) were derived from patients who were temozolomide-naïve at the time of specimen collection (data not shown).

**Table 3:** MGMT deficiency in PDX models relative to temozolomide activity.

PDX Specimen *Diagnosis*	Response to TMZ alone (EFSx4 or CR/PR)	Response to Veliparib + TMZ (EFSx4 or CR/PR)	MGMT promoter status	MGMT protein expression
128128-338-R *Melanoma*	No response	Response	Non-methylated	Negative
172845-121-B *Colon adenocarcinoma*	No response	Response	Methylated	Negative
172845-121-T *Colon adenocarcinoma*	No response	Response	Methylated	Negative
692163-330-T ** Uterine leiomyosarcoma	No response	Response	Non-methylated	Positive
997726-040-R** Lung squamous cell carcinoma	Response	Response	Methylated	Negative
BL0293-F563** Urothelial/bladder cancer	Response	Response	Methylated	Positive & Negative*
BL0382-F1232** Urothelial/bladder cancer	No response	Response	Methylated	Positive
CN0330-F216 *Colon adenocarcinoma*	Response	Response	Methylated	Negative
LG0520-F434 *Lung squamous cell carcinoma*	Response	Response	Non-methylated	Positive
SA0426-F1136 *Non-uterine leiomyosarcoma*	Response	Response	Non-methylated	Positive

Analysis from responsive PDX models based on *MGMT* promoter methylation and MGMT protein expression in tumor samples. IHC positive cutoff: tumor nuclei staining >30% positive. EFSx4 ≥ 2 and/or PR/CR represents responders. For response details (EFSx4 and CR/PR) see **Supplementary Table S2**. *MGMT protein expression changed from positive to negative over 2 consecutive PDX passages. **Models with actionable mutations in the DNA damage repair pathway.

### 3.3 OncoPrint analysis of veliparib and temozolomide responding models

**Figure 3:**
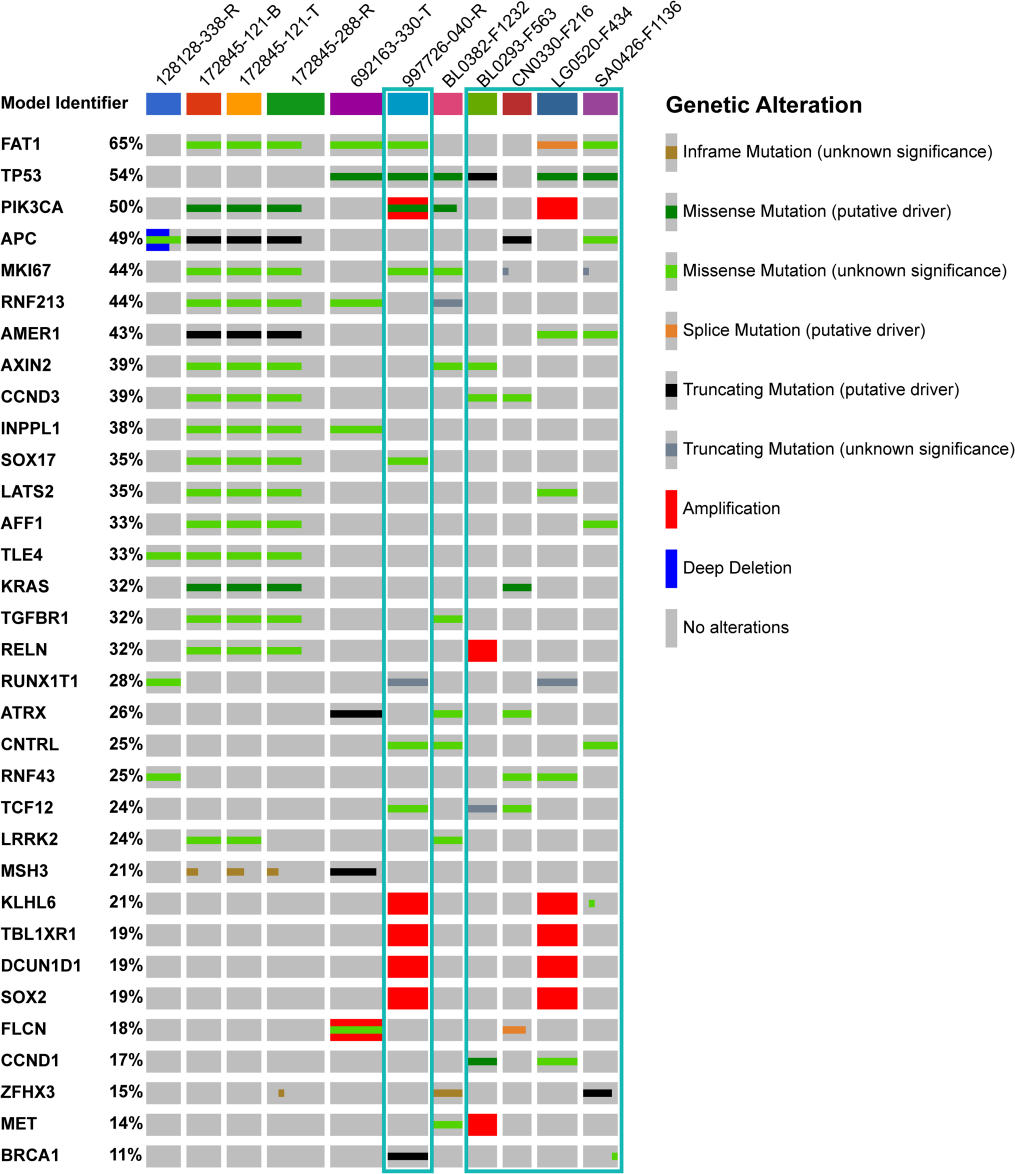
Genes associated with response to veliparib plus temozolomide. OncoPrint map illustrating the most prevalent genetic alterations in the 11 PDX models that responded to veliparib plus temozolomide, as well as genetic alterations in the DNA repair pathway genes of interest for treatment selection in the clinical NCI-MPACT study. A minimum of 5 samples were analyzed per model; column width is determined by the number of samples analyzed. Percentages of samples harboring a genetic alteration are presented next to the gene symbol. Highlighted in teal: models responding to temozolomide (Tmz) as a single agent. Display was ordered based on Sample ID.

An abbreviated OncoPrint map of the most prevalent genomic alterations in the 11 models responding to single agent temozolomide and the combination with veliparib is provided in **Figure 3**. The most frequently altered gene associated with response to this combination was *FAT1* (7/11 PDX models, and 65% of all samples tested). In contrast, only 38% of samples derived from PDX models resistant to the veliparib plus temozolomide combination had a *FAT1* genomic alteration (**Supplementary Figure S1A**). The most frequent genomic alteration in models resistant to the veliparib plus temozolomide combination was detected in the *KMT2D* gene (**Supplementary Figure S1B**); only a single model sensitive to the combination had alterations in the same gene (**Supplementary Figure S1C**).

### 3.4 MGMT promoter methylation status is associated with response to temozolomide

Only 1/11 models (9%) that responded to temozolomide as a single agent or as part of combination treatment harbored an NCI-MPACT aMOI that would have made it eligible for this treatment, highlighting the need to identify additional genomic alterations associated with response. Genomic analysis of the PDX models identified *MGMT* promoter methylation status as a potential predictor of response to temozolomide-based therapy.

Eleven of 49 models tested (22.4%) had a methylated *MGMT* promoter based on a methylation threshold of PMR ≥ 2 (14) (**Supplementary Table S2**). Two of these promoter methylated models were from urothelial/bladder cancers, 7 were colon adenocarcinomas (of which 4 were derived from the same 172845 originator patient), and there was one each of lung squamous cell carcinoma and neuroendocrine cancer (**Table 3**). Calculating the methylation positivity rate based on the number of originator patients, 8/41 patient samples used to generate the PDX models (19.5%) had methylated *MGMT* promoters. All PDX models derived from the same patients had the same *MGMT* promoter methylation status.

**Figure 4:**
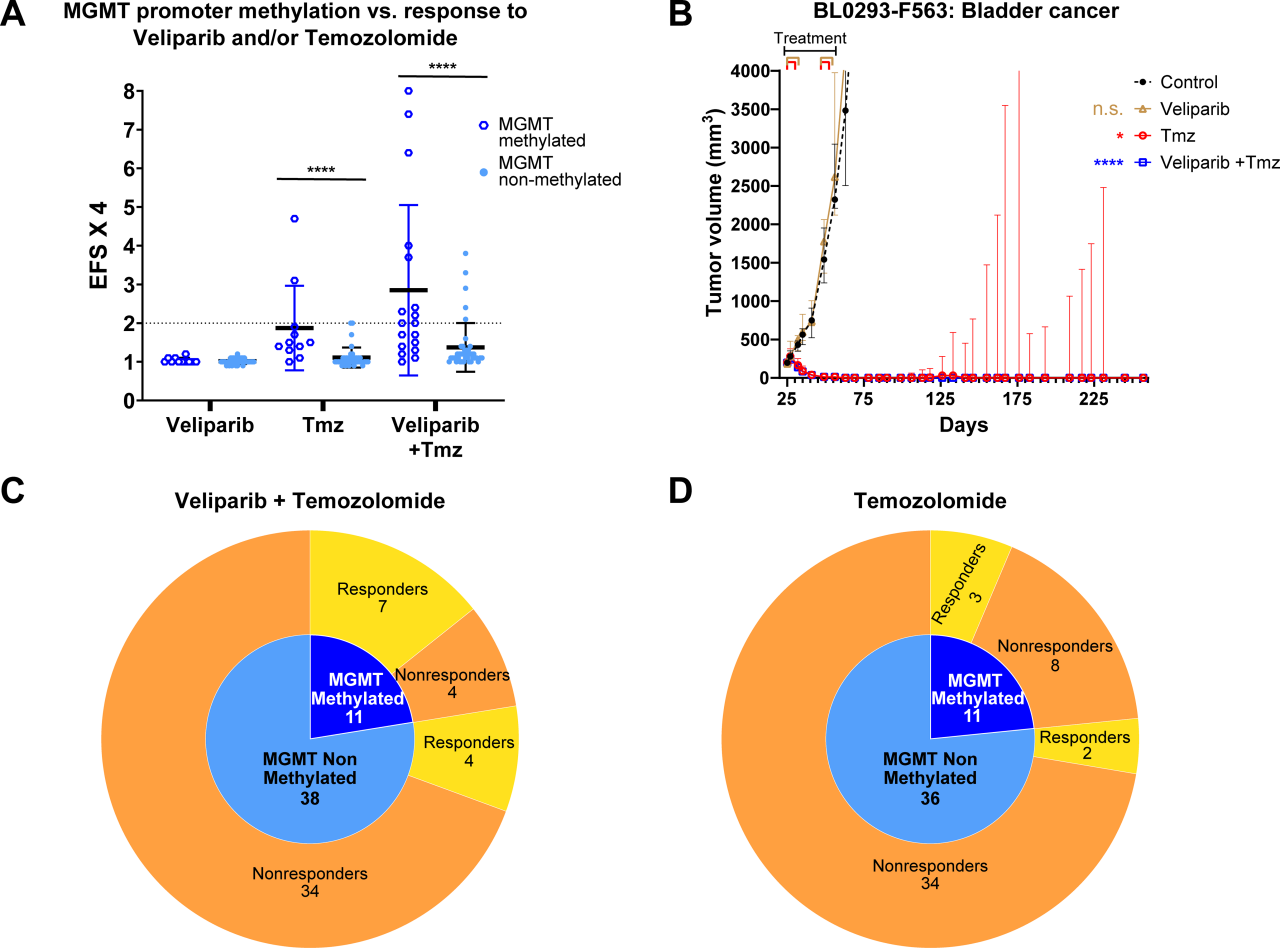
Response to temozolomide-based therapy in the context of *MGMT* promoter methylation. **(A)** Antitumor response to temozolomide (Tmz) and to the combination of veliparib plus temozolomide was significantly greater in PDX tumor models with a methylated *MGMT*-promoter using on EFSx4 ≥ 2 as a threshold (****p < 0.0001; Mann-Whitney test). **(B)** Median tumor volume for BL0293-F563 bladder cancer model with methylated *MGMT* promoter treated with temozolomide, veliparib and combination therapy (temozolomide EFS x 4 = 4.7, PR/CR; veliparib plus temozolomide EFS x 4 = 6.4, PR/CR). Treatment duration indicated on top. Error bars: 95% confidence interval. (**** *p* < 0.0001; Kruskal Wallis analysis and Dunn’s multiple comparison test). **(C)** Pie chart illustrating the higher number of PDX models with a methylated *MGMT*-promoter responding to temozolomide plus veliparib combination compared to models without *MGMT* promoter methylation; EFSx4 ≥ 2 and/or PR/CR as a threshold for response. **(D)** Pie chart illustrating higher number of PDX models with methylated *MGMT*-promoter responding to single agent temozolomide compared to models without *MGMT* promoter methylation; EFSx4 ≥ 2 and/or PR/CR as a threshold for response.

*MGMT* promoter methylation status was subsequently evaluated relative to temozolomide response (**Figure 4A**). Three of 5 models (60%) responding to single-agent temozolomide (EFSx4 ≥ 2 and/or PR/CR) (**Figure 4B**) had a methylated *MGMT* promoter (**Supplementary Table S2**). Four of the 6 (66.7%) remaining models that met response criteria (EFSx4 ≥ 2 and/or PR/CR) for the veliparib plus temozolomide combination, but not temozolomide alone, had methylated *MGMT* promoters (**Figures 4C, D**, **Supplementary Table S2**). Three of these models responding to the combination were derived from the same 172845-colon adenocarcinoma patient originator. Therefore, responders to temozolomide as a single agent or in combination with veliparib were enriched among models with *MGMT* promoter methylation. Although the response rate to temozolomide alone was higher for models with methylated *MGMT* promoters (27% vs 6%), because the sample sizes were small, the difference does not attain statistical significance (2-sided p-value = 0.08, by Fisher’s exact test). The difference between the response rates to the temozolomide plus veliparib combination in models with *MGMT* methylated vs non-methylated promoters (64% vs 11%) is statistically significant despite the small sample size (2-sided p-value = 0.001, by Fisher exact test). As indicated above, there were 51 models taken from 43 unique patients. Limiting the sample to 1 model from each of the unique patients would have involved an arbitrary elimination of 8 models and it is unlikely that it would have meaningfully changed the results. Representative models with methylated *MGMT* promoter that responded to temozolomide-based therapy, and non-responsive models without *MGMT* deficiency are illustrated in **Supplementary Figure S3**.

MMR deficiency, specifically loss of mismatch repair proteins such as MLH1, MSH2, MSH3, MLH3, MSH6 and PMS2, can decrease the cytotoxicity of temozolomide treatment in MGMT-deficient cells (6). An overview of genomic alterations of MMR genes in MGMT-deficient models is presented in **Supplementary Figure S2A**. Two of the 4 PDX models with *MGMT* promoter methylation (625472-104-R and 997537-175-T, both colon adenocarcinomas) that did not respond to either single agent temozolomide or the combination with veliparib had very low *MLH1* mRNA levels (log2[normalizedCount+1] < 4). These same non-responsive models also expressed lower levels of *MSH2* and *MSH3* mRNA compared to models that responded to either temozolomide single treatment and/or the combination (**Supplementary Figure S2B**); however, these models also expressed higher levels of *MGMT* mRNA compared to other models having *MGMT* promoter methylation. These data suggest that response to temozolomide-based therapy may be associated with MGMT deficiency and MMR proficiency.

### 3.5 MGMT protein expression in MPACT PDX models

MGMT protein expression and the stability of expression over consecutive passages was assessed by IHC in samples from 49 PDX models; a cutoff of > 30% nuclei staining was employed for positive samples (**Table 3**). Eighteen of the models were derived from treatment-naïve patients, 27 models were derived from patients who had received clinical therapy other than temozolomide; 4 models were derived from patients with unknown treatment history. Six of the 49 (12.2%) models did not express MGMT. Lack of MGMT protein expression correlated with *MGMT* promoter methylation (5/11 models, 45%); a single model (128128-338-R) without *MGMT* promoter methylation lacked MGMT protein expression. A seventh model— BL0293-F563 bladder cancer—changed from positive to negative between passages 4 and 5 (**Table 3**, **Supplementary Figure S4A**).

### 3.6 MGMT RNA expression across PDX models in the PDMR

*MGMT* RNA expression across 5348 specimens derived from 1056 PDX tumor models in the PDMR shows a bimodal expression profile with ~ 10% of the samples across several different tumor types having no detectable *MGMT* mRNA (**Supplementary Figure S4B**; **Supplementary Table S3**). Low *MGMT* mRNA expression was not due to mRNA sample degradation, as there was no correlation between the total number of RNA reads and of *MGMT* mRNA reads (**Supplementary Figure S4C**). Concordance between *MGMT* promoter methylation status and *MGMT* RNA-seq data in the preclinical MPACT models are shown in **Supplementary Figure S4D**. A breakdown of *MGMT* RNA reads per type of cancer is available in **Supplementary Figure S4E**. All 6 models lacking MGMT expression in 1 or 2 tested passages also had low levels of corresponding mRNA as measured by RNA-seq (5 had an average RSEM z-score < -2; 172845-121-B and 128128-338-R had an average z-score < -1.8).

## 4 Discussion

To our knowledge, preclinical NCI-MPACT is the largest randomized basket PDX study evaluating response to targeted anticancer therapy. It used a large cohort of well characterized patient-derived tumor material to examine whether distinct allele frequencies influence response to therapy in heterogenous tumors, to evaluate the potential specificity of the aMOIs studied with respect to tumor response, and to determine novel genomic and molecular mechanisms behind responses (19, 20). Evaluating clinically relevant parameters of xenograft model response is challenging (11). In this study, we used two independent metrics to quantify response: EFSx4 to provide a quantifiable and consistent metric of growth delay, as well as clinically relevant metrics measuring tumor regression (11, 21).

The NCI-MPACT clinical trial was marked by a lack of clinical activity in the targeted treatment cohorts: one patient had an objective response to trametinib; 7 patients across the targeted treatment arms had stable disease lasting more than 8 cycles (1). Accrual was halted early in the veliparib plus temozolomide arm because every patient with a DNA repair pathway aMOI possessed a *TP53* mutation believed to be insensitive to veliparib and temozolomide (1). In part, the response rate observed on this companion preclinical study confirms the findings from the clinical trial. There was little correlation between the presence of actionable mutations and response (either ESFx4 ≥ 2, and/or PR/CR) to specifically-targeted treatment in this preclinical study. Two PDX models responded to trametinib (one lacking an aMOI conferring clinical study eligibility; one harboring an actionable KRAS mutation); none of the 6 models harboring a BRAF (V600E) mutation responded to trametinib, despite the lack of prior MEK inhibitor therapy in the originator patients. Two models, neither with an aMOI, responded to adavosertib plus carboplatin; 11 (22%) models responded to veliparib plus temozolomide, of which only 1/11 (9%) models harbored an aMOI that would have made it eligible for the treatment; no model responded to everolimus (**Figure 2A**, **Supplementary Table S2**).

Eighteen of the 51 models (35.3%) harbored mutations in more than one genetic pathway, of which 5 models would have made the patients who donated the tumor material eligible for more than 1 NCI-MPACT targeted treatment based on variant abundance. None of these models with multiple aMOIs responded to targeted treatment, which can be attributed to genomic heterogeneity in these advanced, treatment refractory tumors. Model and lineage heterogeneity was also explored in 11 models generated from 3 patients and derived from different longitudinal timepoints and anatomic locations. Seven of the models (derived from 2 patients) had similar responses to both targeted and non-targeted treatment. In models derived from patient 172845 (colon adenocarcinoma) that shared a similar mutation profile and had the same *MGMT* promoter methylation status, response to temozolomide-based therapy varied based on the MGMT protein expression (**Supplementary Table S2**), indicating that transcriptional and post-transcriptional processes also influence response to therapy.

The most clinically relevant result from this prospective preclinical study was the identification of temozolomide as a critical component of the response to the combination with veliparib recorded in nearly 20% of the models, (**Figures 2C**, **4**, **Table 1**, **Supplementary Table S2**). PARP inhibition potentiated the response of temozolomide (22, 23) as suggested by the higher number of models that responded to the combination compared to single agent temozolomide treatment (**Figure 2C**). Temozolomide is currently only approved by the FDA for patients with glioblastoma multiforme (GBM) or anaplastic astrocytoma, but response to temozolomide is associated with MGMT deficiency and a functional DNA MMR pathway in several different cancer types (3–6). We therefore assessed MGMT deficiency in the models by measuring *MGMT* promoter methylation status, mRNA levels, and protein levels to correlate with response to temozolomide. *MGMT* promoter methylation status has previously been reported to be the more reliable predictor of temozolomide response compared to protein or mRNA measurements in GBM (24). Eleven of the 49 PDX models tested (22.4%) had a methylated *MGMT* promoter; 5 of these 11 models tested did not express MGMT protein, and a sixth model lost MGMT expression at later passages (**Supplementary Figure S4A**), suggesting that MGMT expression is not always stable. The low correlation between *MGMT* promoter methylation status and MGMT protein expression in our study is confirmed by previous reports (24). BL0479-F1894 was the only model lacking MGMT protein expression that did not respond to temozolomide. This model also lacked an NCI-MPACT aMOI suggesting that additional genomic, transcriptomic, or post-translational alterations conferring temozolomide sensitivity or resistance are yet to be identified. *MGMT* promoter methylation status associates with mRNA expression in our data set (p<0.0001, two-tailed t-test) (**Supplementary Figure S4D**), as previously reported (25); however, the clinical utility and applicability of RNA-seq methods to stratify patients likely to benefit from temozolomide remain to be tested.

That six of 11 models (54.4%) with a methylated *MGMT* promoter and 5/6 models (83.3%) lacking MGMT protein expression responded to temozolomide or the combination, indicates that MGMT deficiency represents a more useful biomarker for assignment to temozolomide-based therapies than the aMOIs in the NCI-MPACT study. The greater number of PDX models benefiting from temozolomide based on promoter methylation status suggests that selection of MGMT-deficient patients using IHC alone may exclude subjects who could benefit from this drug. Importantly, previous studies have confirmed modest response rates to temozolomide given as a single agent in colorectal cancers with *MGMT* promoter methylation (26, 27), and to temozolomide-based regimens in pancreatic neuroendocrine tumors lacking MGMT protein expression (28), supporting our observations that MGMT deficiency may be a predictor of response to temozolomide regardless of tumor histology.

Several assays for measuring MGMT deficiency in tumor samples have been developed and clinically evaluated (15, 29, 30). While all these assays have research value, *MGMT* promoter methylation allows for higher-throughput evaluation. We therefore developed and validated an extremely sensitive promoter methylation assay which has a very high negative predictive value necessary for its intended use as a CLIA high-throughput screen of patients in our clinic (**Supplementary Materials**). However, the positive predictive value of our assay could not be accurately determined due to the small number of responders on our preclinical study, and it is important to keep in mind that the high sensitivity to temozolomide reported in PDX models may not be replicated in the clinic due to species differences in pharmacokinetics (31). However, based on our observations in this preclinical study, combining *MGMT* promoter methylation and a sensitive IHC to confirm lack of MGMT expression may hold superior value when identifying patients likely to benefit from temozolomide-based therapies.

Touny et al. reported that 31% of *MGMT*-low and MMR-proficient patient-derived and established cancer lines are sensitive to temozolomide at clinically relevant concentrations (32). Combination treatment with ATR inhibitors (32, 33), base excision repair inhibitors (7), or treatment with immune checkpoint inhibitors following temozolomide priming (34) might overcome temozolomide resistance in this circumstance.

Of interest was the finding that *FAT1* was mutated in more than half of the models responding to temozolomide single and/or combination treatment. FAT1 is a transmembrane protein belonging to the cadherin family that has been shown to regulate cell-cell interactions, actin cytoskeleton dynamics and cellular migration in both healthy and cancer cells (35–37). Depending on the tumor type, FAT1 has been shown to function as both an oncogene promoting HIF1α expression, invasiveness (36), and inflammation in advanced tumors (38), as well as a tumor suppressor by inhibiting Wnt signaling and tumorigenesis (35). FAT1’s role in the context of response to temozolomide-based therapies remains to be investigated.

Preclinical studies allow each model to be treated with multiple agents or combinations, allowing sufficient statistical power and precision to identify genomic characteristics predictive of treatment response, the superiority of different treatments, and the contribution of each drug in a combination regimen. Would the NCI have conducted the clinical MPACT trial if the preclinical study had been completed first? We would have modified the study design to reflect the high prevalence of patients with TP53 mutations. In the clinical study, patients with *TP53* aMOIs were assigned to the adavosertib plus carboplatin arm rather than veliparib plus temozolomide based on preclinical data that loss-of-function mutations in *TP53* affect regulation of cell cycle progression (1). Veliparib was selected based on our experience with this agent and its favorable safety profile, but a stronger PARP-trapping agent might have been considered (39, 40). We are also more cognizant now that aMOIs in single driver genes are not always determinative of clinical response (41). The NCI’s current precision medicine targeted agent trial, comboMATCH, requires supportive evidence from xenograft studies to determine whether the presence of known tumor genomic variants can predict clinical activity (41).

The prospective nature of our study allowed us to identify in an unbiased manner markers for temozolomide response in a broad range of solid tumors. The data suggest that patients with MGMT deficiency harboring a variety of malignancies might benefit from temozolomide single-agent or combination therapies. This study also underscores the value of using well characterized and adequately controlled patient-derived models to identify molecular signatures that could inform clinical trials.

## Data Availability

The original contributions presented in the study are included in the article/**Supplementary Material**. Further inquiries can be directed to the corresponding author.
