# Supplementary material for: Preclinical NCI-MPACT: prospective modeling of the mutation-based NCI-MPACT clinical trial therapeutic strategy in patient-derived xenograft models
Source: Front Oncol. 2025 May 19;15:1571635. doi: 10.3389/fonc.2025.1571635 (PMC12127203; doi:10.3389/fonc.2025.1571635)
Supplement: Supplementary file 1 [file DataSheet1.docx]

***Supplementary Material for Preclinical NCI-MPACT: Prospective Modeling of the Mutation-Based NCI-MPACT Clinical Trial Therapeutic Strategy in Patient-Derived Xenograft Models***

# Supplementary Methods

## Whole Exome Sequencing

Whole exome libraries from each sample were generated by shearing 50 ng genomic DNA to 150–180 bp using Covaris LE220 sonicator (Covaris Inc., Woburn, MA, USA).  The library preparation procedure was automated on Perkin Elmer SciClone G3 platform using custom scripts (Perkin Elmer Inc., Waltham, Ma, USA).  The sheared genomic DNA was processed using Kapa Hyper library construction and dual index kit through end-repair, dA tailing, ligation with indexed Illumina adaptors (Roche Sequencing and Life Science, Indianapolis, IN, USA), based on Kappa Biosystems recommendations.  The adaptor ligated libraries were purified using AMpure XP beads (Beckman Coulter, CA, USA), amplified by PCR (12 cycles for germline samples, four cycles for tumor FFPE samples) using KAPA HiFi polymerase, and subsequently re-purified (AMpure XP beads).  Seven hundred and fifty ng of the purified amplified libraries were hybridized with a biotinylated RNA bait set (SureSelectXT Human V5, Agilent Technologies Inc., CA, USA) at 65°C for 16 hours.  The captured genomic DNA fragments were enriched by Dynal MyOne Streptavidin T1 beads (Thermo Fisher Scientific, MA, USA.) and amplified for 12 PCR cycles using Hercules DNA polymerase (Agilent Technologies Inc., CA, USA).  The amplified libraries were purified using AMPure XP beads, checked for size distribution (300–400 bp) using Agilent TapeStation 4200 (Agilent Technologies Inc., CA, USA) and quantified using BioRad ddPCR Library Quantification Kit for Illumina TruSeq (BioRad Laboratories, CA, USA).  Pooled libraries were prepared by mixing final libraries at equal molar ratio and quantified using BioRad ddPCR Library Quantification Kit for Illumina TruSeq (BioRad Laboratories, CA) in a BioRad QX200 digital PCR system.  The final quantitated pooled libraries were diluted to 1.2 nM (for XP mode S4 flowcell workflow) (Illumina Inc., San Diego, CA, USA).  The sequencing was performed on Illumina NovaSeq 6000 using 2 x 150 bp paired-end sequencing mode.

## Whole Transcriptome Sequencing

RNASeq library preparation procedure was automated on the Perkin Elmer SciClone G3 platform using custom scripts.  RNASeq libraries were prepared using 10–70 ng of total fragmented RNA, followed by cDNA synthesis.  Purified cDNA (AMpure XP beads) was processed through dA tailing, ligation with indexed adaptors using Illumina Truseq RNA Exome kit (Illumina Inc., CA, USA) and re-purified.  The libraries were subsequently enriched by PCR amplification (15 cycles).  The quality and quantity of the purified enriched libraries were assessed using D1000 tape in Agilent TapeStation.  Two hundred nanograms of purified amplified libraries were subject to two consecutive hybridizations with a biotinylated DNA bait set, captured with streptavidin magnetic beads, washed and eluted (Illumina RNA Exome, Illumina Inc., San Diego, CA, USA) following the vendor’s recommended protocol.  The enriched libraries were purified (AMpure XP beads), amplified (10 PCR cycles), and re-purified (AMpure XP beads) to obtain the final RNASeq libraries.  The quality and quantity of the final libraries were assessed by Agilent Tapestation and Molecular Dynamics Spectramax fluorometer (Molecular Dynamics, CA, USA), respectively.  The final libraries were prepared and sequenced as described in the WES methods section. An equimolar pool of final libraries was prepared and quantitated using the BioRad ddPCR Library Quantitation kit on BioRad QX200 digital PCR system and diluted to 1.2 nM (for XP mode of clustering on NovaSeq S4 flowcell).  Sequencing was performed on Illumina NovaSeq 6000 using 2 x 150 bp paired-end sequencing mode.

## Bioinformatics data analyses for WES and RNASeq datasets

For each flowcell from Illumina HiSeq2000 or HiSeq2500 or NovaSeq run, .BCL files were used for base calling and converted to demultiplexed FASTQ files using the bcl2fastq tool.  FASTQ files were then run through FastQC (http://www.bioinformatics.babraham.ac.uk/projects/fastqc/) for quality checking.  For PDX specimens, mouse reads were removed from the raw FASTQ files using bbsplit (bbtools v37.36) (https://sourceforge.net/projects/bbmap) by mapping reads to human and mouse genome and FASTQ files containing human-only reads were generated; ambiguous reads were also retained.

For WES data, human-only reads FASTQ files were mapped against human genome reference hg19 using bwa-mem (Burrows-Wheeler Alignment Tool) (1).  The resulting bam file was sorted and indexed using Sentieon tools (Sentieon Inc., San Jose, CA) (sentieon-genomics/201711.01)(2); GATK Best Practices were implemented to mark duplicates, generate local realignment and recalibrate base quality score (3)).  Depending on the availability of the paired normal specimen, either tumor-only or paired tumor-normal variant caller options were implemented.  For the tumor-only mode, genomic variants were called by Sentieon Haplotyper (Sentieon implementation of GATK HaplotypeCaller) and Platypus (4). Merged VCF files were generated from the results of 2 variant callers.  For paired tumor-normal mode, somatic mutations (SNVs and indels) were called by Sentieon TNhaplotyper (GATK Mutect2 implementation in Sentieon tools, [Sentieon Inc., San Jose, CA]).

For RNASeq data, the human-only reads FASTQ files are mapped to the human transcriptome based on exon models from hg19 using Bowtie2 (version 2.2.6) (5). The resulting SAM files are converted to BAM format using samtools [2], and the genomic coordinates in BAM are converted to the genomic (hg19) coordinates using RSEM (version 1.2.31) (6). Gene and transcript quantifications were generated using RSEM.

## Methylation-specific PCR assay for MGMT promoter methylation

Briefly, DNA was extracted from formalin-fixed, paraffin embedded (FFPE) tissues using the Qiagen FFPE DNA/RNA Kit as described; DNA (approximately 50 ng) was converted from unmethylated cytosine residues into uracil residues using sodium bisulfite treatment (Qiagen EpiTect Bisulfite Kit). Real-time PCR methylation analysis was performed using the MethyLight assay with methylation-specific primers for sensitive discrimination occurring at the PCR amplification level (Qiagen EpiTect MethyLight PCR + ROX Vial Kit). To evaluate the relative methylation level, the percentage of methylated reference (PMR) was calculated for each sample by dividing the quantity mean of MGMT sample/methylated positive control by the quantity mean of ACTB sample/methylated positive control (Applied Biosystems QuantStudio ViiA7 software). PMR was calculated based on the formula:

$$PMR=100\times\frac{Qsample MGMT}{QMethylPosCtrl MGMT}\div\frac{Qsample ACTB}{QmethylPosCtrl ACTB}$$

For clinical applications, the limit of reporting has been established at ≥3% methylation with a positive reporting threshold of PMR ≥2.

***MGMT promoter methylation assay validation***

Analytical validation of the *MGMT* Promoter Methylation Assay, was performed by the CLIA-certified Molecular Characterization Laboratory (MoCha) at the Frederick National Laboratory for Cancer Research. For validation results, see **Supplemental Tables V (1-5)** at the end of the Supplemental Methods. Acceptable assay performance criteria were established based on the intended use of the assay and preliminary performance results of a pilot feasibility test; assay validation data are presented below. Multiple clinical formalin-fixed paraffin-embedded (FFPE) glioblastoma and colorectal carcinoma tissue samples were chosen to test the assay performance of the Qiagen EpiTect Bisulfite Kit and the Qiagen EpiTect MethyLight PCR +ROX Vial Kit, a TaqMan real-time PCR assay that uses primers and probes designed for the methylated *MGMT* and *ACTB* genes. DNA was quantified and samples sufficient for assay performance were then subjected to bisulfite treatment as described above. To evaluate the relative methylation level, the percentage of methylated reference (PMR) was calculated for each sample as described above. Standard curve QC metrics, positive and negative control QC metrics, and PMR calculation by designated laboratory personnel were manually reviewed to confirm adequate PCR efficiency and quality. **Supplemental Table M1** lists acceptable assay performance criteria established in the validation plan.

**Supplemental Table M1**: Summary of Acceptance Criteria for the *MGMT* promoter methylation assay

| **Acceptance criteria:** | **Overall** |
| --- | --- |
| Limit of detection | ≥ 5% methylation |
| Sensitivity | ≥ 93% |
| Specificity | ≥ 93% |
| Reproducibility (intra-operator) | ≥90% |
| Reproducibility (inter-operator) | ≥90% |

*Analytical validation results*

As defined by the approved SOPs, 50 ng of controls and samples were used for all validation studies and all results passed the set QC metrics. Millipore’s Universal Methylated DNA, was used to generate the PCR standard curve at five dilution points, ranging from 12.5 ng to 0.78 ng (**Supplementary Figure M1**). PCR efficiency for *MGMT* and *ACTB* on all runs was between 80–120% and the R^2^ for all standards was >0.9 (**Supplementary Table V1)**. The *ACTB* Ct values for all standards, controls, and clinical specimen were within the expected range. Additionally, all positive, negative, and non-template controls yielded results as expected.

**Supplementary Figure M1:** PCR standard curve for *ACTB* and *MGMT* evaluating PCR efficiency.

*Limit of detection*

To determine the limit of detection (LOD) of the *MGMT* promoter methylation assay, FFPE tumor cell lines from the NCI-60 cell line panel with known methylation status were tested at different dilutions. The methylated cell line K562 was diluted into the unmethylated cell line RKO to approximate methylation (M) percentage at 100% M (K562), 100% Unmethylated (U) (RKO), 50% M (K562 + RKO), 30% M, 20% M, 10% M, 5% M, 4% M, 3% M, 2% M, and 1% M. Each dilution was assayed 10 times and the PMR was calculated.

Results of the LOD study are listed in **Supplemental Table M2**. PMR calculations for each dilution point across the 10 replicates are shown, with PMR ≥2 values detected consistently for all sample replicates with a 3% methylation rate or higher. PMR < 2 were detected for 2 and 4 replicates at 2% and 1% methylation, respectively.

**Supplemental Table M2:** Limit of detection of the *MGMT* promoter methylation assay **(PMR)**

| **Samples** | **Rep1** | **Rep2** | **Rep3** | **Rep4** | **Rep5** | **Rep6** | **Rep7** | **Rep8** | **Rep9** | **Rep 10** | **Mean** | **Std** |
| --- | --- | --- | --- | --- | --- | --- | --- | --- | --- | --- | --- | --- |
| **100%M** | 102.3 | 94.9 | 89.4 | 88.1 | 115.3 | 106.8 | 88.2 | 98.3 | 112.8 | 82.5 | 97.9 | 11.2 |
| **100%U** | 0.0 | 0.0 | 0.0 | 0.0 | 0.0 | 0.0 | 0.0 | 0.0 | 0.0 | 0.0 | 0.0 | 0.0 |
| **50%M** | 50.4 | 53.2 | 42.8 | 45.5 | 68.5 | 66.9 | 61.1 | 62.7 | 76.0 | 61.6 | 58.9 | 10.6 |
| **30%M** | 31.3 | 35.1 | 30.5 | 25.5 | 44.8 | 42.9 | 26.7 | 26.9 | 39.5 | 43.2 | 34.6 | 7.5 |
| **20%M** | 20.4 | 24.1 | 21.7 | 17.1 | 32.6 | 24.0 | 27.9 | 20.1 | 28.7 | 27.8 | 24.4 | 4.8 |
| **10%M** | 8.8 | 13.0 | 9.0 | 6.0 | 18.4 | 17.6 | 11.3 | 11.4 | 15.3 | 14.7 | 12.6 | 4.0 |
| **5%M** | 4.5 | 4.7 | 4.5 | 4.5 | 10.3 | 7.8 | 5.4 | 3.9 | 9.8 | 5.5 | 6.1 | 2.3 |
| **4%M** | 3.8 | 4.7 | 4.0 | 4.7 | 7.2 | 7.3 | 4.8 | 4.4 | 5.4 | 3.9 | 5.0 | 1.3 |
| **3%M** | 2.9 | 4.4 | 3.6 | 2.4 | 8.0 | 4.9 | 3.5 | 2.6 | 3.8 | 4.0 | 4.0 | 1.6 |
| **2%M** | 1.1 | 3.3 | 2.3 | 1.3 | 4.3 | 4.0 | 3.0 | 3.9 | 4.0 | 2.8 | 3.0 | 1.1 |
| **1%M** | 1.7 | 2.4 | 2.2 | 2.2 | 1.0 | 3.2 | 2.7 | 2.0 | 1.1 | 2.6 | 2.1 | 0.7 |
| **0.5%M** | 0.0 | 1.5 | 1.4 | 0.6 | 0.6 | 0.4 | 1.1 | 0.6 | 0.8 | 0.7 | 0.7 | 0.5 |

Additionally, 10 replicates of 0.5% methylated samples and 3 replicates of the 0.25% methylated samples were tested in triplicates to determine if amplification is detected below 1% methylation. This assay was able to detect methylation in only two out of 9 wells of the 0.25% methylated sample. The calculated limit of detection of this *MGMT* promoter methylation assay is therefore 0.5% methylation, superior to the acceptance criteria of 5%. However, for clinical applications the limit of reporting has been established at ≥3% methylation with a reporting threshold of PMR ≥2. Samples with results lower than PMR of 2 are reported as negative.

*Analytical sensitivity*

To assess sensitivity, 15 well-characterized and known *MGMT* methylated clinical samples that were previously confirmed as *MGMT*-positive by Dartmouth-Hitchcock’s analytically validated assay (7) were tested on our *MGMT* promoter methylation assay. Information on assay validation and quality metrics were provided to support the validity of the methylation status in the selected specimens as shown in **Supplementary Table V2**.

The results were assessed as methylation positive or methylation negative, based on the PMR threshold of 2. A total of 14 samples had a PMR ≥ 2 and were considered methylated positive. A single sample (MGMT-11) had a PMR of 0.9; the value was below the reporting threshold and recorded as “not detected.” The *MGMT* promoter methylation assay therefore met the required acceptance criteria of ≥93%. Detailed information for each sample’s PMR reported by the *MGMT* promoter methylation assay is listed in **Supplementary Table V3**.

*Analytical Specificity*

Specificity of the *MGMT* promoter methylation assay was assessed by testing 15-well characterized clinical samples with unmethylated *MGMT* promoter status (**Supplementary Table V2**) previously identified by Dartmouth-Hitchcock’s analytically validated assay (7). Information on assay validation and quality metrics were provided to support the validity of the methylation status in the selected specimens as shown in **Supplementary Table V3**.

The results were assessed as methylation-positive or methylation-negative, based on the PMR threshold of 2. All 15 samples had a PMR value of 0 and were considered methylation-negative. The assay therefore did not detect any false positives, resulting in 100% specificity for each sample, meeting the acceptance criteria. Detailed information for each sample is listed in **Supplementary Table V4**.

*Analytical Reproducibility*

A reproducibility study was performed using 4 replicates of 10 samples divided into 5 methylation-positive and 5 methylation-negative samples; each reproducibility test was a technical replicate assayed by 2 different operators on different days starting from aliquots of nucleic acid that were blinded to both operators. Of the 10 specimens, and across the 4 replicates for each, 5 samples resulted in PMR ≥2 indicating methylation-positive, and 5 samples resulted in PMR of 0 indicating methylation-negative.

The *MGMT* promoter methylation assay showed 100% agreement for all reproducibility runs, for both intra-operator and inter-operator analysis, meeting the acceptance criteria of greater than 90% agreement. Detailed information for each sample’s PMR reported by the *MGMT* promoter methylation assay is listed in **Supplementary Table V5**.

*Summary and Conclusions*

As shown in **Supplemental Table M3**, the *MGMT* promoter methylation assay meets the acceptance criteria defined above in the Validation Plan for analytical performance and the data demonstrate that the assay is suitable for its intended use in clinical testing: the assay demonstrated an overall sensitivity of 93.33%, overall specificity of 100%, and an overall reproducibility of 100%.

**Supplemental Table M3**: Final acceptance criteria and outcomes

| **Criterion** | **Required** | **Actual** | **Outcome** |
| --- | --- | --- | --- |
| Limit of Detection (%MGMT) | ≥5% | ≥0.5% | Pass |
| Limit of Reporting (%MGMT) | - | ≥0.3% | - |
| Limit of Reporting (PMR) | - | ≥2 | - |
| Sensitivity | ≥93% | 93.33% | Pass |
| Specificity | ≥93% | 100% | Pass |
| Reproducibility (Intra-operator) | ≥93% | 100% | Pass |
| Reproducibility (Inter-operator) | ≥93% | 100% | Pass |

*Thermal cycler bridging study*

The PMR data was consistent and comparable between two tested thermal cycler models (ABI Veriti and Bio-Rad C1000), thus indicating that methylation status reporting does not change with the brand of cycler used.

*Concordance with pyrosequencing assay*

To assess concordance of the pyrosequencing assay (8) with our *MGMT* promoter methylation assay, 50 clinical GBM specimens (25 positive and 25 negative) with corresponding pyrosequencing methylation data were tested. All 25 negative samples had a PMR of <2 on our *MGMT* promoter methylation assay, indicating concordant negative results with the negative percent agreement (NPA) 100%. From the 25 positive samples, 7 samples resulted in PMR <2 also indicating negative methylation, making the positive percent agreement (PPA) 72% and an overall percentage agreement (OPA) 86%. Discrepancies were associated with those samples classified as either weak positive (n=2) or those with C/T ratio around 30 (n=5) by the pyrosequencing assay. This is likely due to the differences in CpG sites interrogated, heterogeneity in methylation pattern, and/or differences in detection and quantitation methods. Overall, these results indicate that the *MGMT* promoter methylation assay is concordant with the pyrosequencing assay at a level reported in literature.

**Supplementary Table V1:** Standard Curve Data

| **Experiment** | **MGMT R^2^** | **MGMT efficiency** | **ACTB R^2^** | **ACTB efficiency** |
| --- | --- | --- | --- | --- |
| LOD-1 | 0.903 | 103.51% | 0.998 | 84.53% |
| LOD-2 | 0.955 | 86.33% | 0.996 | 97.39% |
| LOD-3 | 0.966 | 91.86% | 0.991 | 93.17% |
| LOD-4 | 0.948 | 95.46% | 0.998 | 101.12% |
| LOD-5 | 0.951 | 83.47% | 0.994 | 97.76% |
| LOD-6 | 0.92 | 83.63% | 0.997 | 96.63% |
| LOD-7 | 0.985 | 94.84% | 0.991 | 96.30% |
| LOD-8 | 0.989 | 102.74% | 0.998 | 97.05% |
| LOD-9 | 0.99 | 89.73% | 0.995 | 100.82% |
| LOD-10 | 0.983 | 97.86% | 0.993 | 93.59% |
| LOD-11 (0.5%M) | 0.992 | 102.24% | 0.995 | 94.98% |
| LOD-12 (0.5%M) | 0.992 | 100.06% | 0.99 | 96.60% |
| Sensitivity Set 1 | 0.916 | 99.05% | 0.988 | 99.34% |
| Sensitivity Set 2 | 0.985 | 99.12% | 0.99 | 94.08% |
| Specificity Set 1 | 0.959 | 114.94% | 0.997 | 98.17% |
| Specificity Set 2 | 0.992 | 102.13% | 0.995 | 95.89% |
| Reproducibility Set 1 | 0.972 | 102.20% | 0.997 | 104.01% |
| Reproducibility Set 2 | 0.992 | 101.82% | 0.988 | 98.66% |
| Reproducibility Set 3 | 0.994 | 99.17% | 0.995 | 94.07% |
| Reproducibility Set 4 | 0.989 | 85.50% | 0.992 | 82.97% |

**Supplementary Table V2**: Clinical Samples from Dartmouth-Hitchcock

| **Item** | **Sample ID** | **Sample Quant**  **(ng/mcL)** | **Sample**  **Volume (mcL)** | **Purpose of**  **Shipment** | **Results** | **PMR Values** |
| --- | --- | --- | --- | --- | --- | --- |
| 1 | MGMT 1 | 80.0 | 5 | Assay Validation | Positive | 21.1, 21.1 |
| 2 | MGMT 2 | 56.4 | 5 | Assay Validation | Positive | 16.6, 16.6 |
| 3 | MGMT 3 | 88.0 | 5 | Assay Validation | Positive | 33.7, 27.8 |
| 4 | MGMT 4 | 50.8 | 5 | Assay Validation | Positive | 11.9, 11.2 |
| 5 | MGMT 5 | 74.2* | 5 | Assay Validation | Positive | 46.2, 29.5 |
| 6 | MGMT 6 | 66.4 | 5 | Assay Validation | Positive | 20.1, 19.5 |
| 7 | MGMT 7 | 73 | 5 | Assay Validation | Positive | 3.9, 3.9 |
| 8 | MGMT 8 | 76.4 5 | 5 | Assay Validation | Positive | 41.5, 48.1 |
| 9 | MGMT 9 | 103.6 | 5 | Assay Validation | Positive | 50.7, 48.7 |
| 10 | MGMT 10 | 59 | 5 | Assay Validation | Positive | 53.0, 60.8 |
| 11 | MGMT 11 | 50 5 | 5 | Assay Validation | Positive | 3.5, 3.5 |
| 12 | MGMT 12 | 50.8 | 5 | Assay Validation | Positive | 28.2, 28.2 |
| 13 | MGMT 13 | 204 | 5 | Assay Validation | Positive | 13.3, 12.3 |
| 14 | MGMT 14 | 183 | 5 | Assay Validation | Positive | 36.0, 33.4 |
| 15 | MGMT 15 | 89.6 | 5 | Assay Validation | Positive | 49.0, 52.1 |
| 16 | MGMT 16 | 110 | 5 | Assay Validation | Positive | 15.4, 14.3 |
| 17 | MGMT 17 | 64.2 | 5 | Assay Validation | Positive | 19.9, 15.4 |
| 18 | MGMT 18 | 114 | 5 | Assay Validation | Positive | 15.3, 11.8 |
| 19 | MGMT 19 | 216 | 5 | Assay Validation | Positive | 13.4, 13.0 |
| 20 | MGMT 20 | 142 | 5 | Assay Validation | Positive | 14.9, 14.5 |
| 21 | MGMT 21 | 113.5 | 5 | Assay Validation | Negative | 0.05, 0.05 |
| 22 | MGMT 22 | 242.1 | 5 | Assay Validation | Negative | 0.0, 0.0 |
| 23 | MGMT 23 | 89.4 | 5 | Assay Validation | Negative | 0.05, 0.05 |
| 24 | MGMT 24 | 81.6 | 5 | Assay Validation | Negative | 0.0, 0.0 |
| 25 | MGMT 25 | 97.2 | 5 | Assay Validation | Negative | 0.0, 0.0 |
| 26 | MGMT 26 | 95.4 | 5 | Assay Validation | Negative | 0.0, 0.0 |
| 27 | MGMT 27 | 97.4 | 5 | Assay Validation | Negative | 0.01, 0.0 |
| 28 | MGMT 28 | 162 | 5 | Assay Validation | Negative | 0.0, 0.0 |
| 29 | MGMT 29 | 102 | 5 | Assay Validation | Negative | 0.0, 0.0 |
| 30 | MGMT 30 | 114.4 | 5 | Assay Validation | Negative | 0.02, 0.02 |
| 31 | MGMT 31 | 228 | 5 | Assay Validation | Negative | 0.0, 0.0 |
| 32 | MGMT 32 | 101 | 5 | Assay Validation | Negative | * |
| 33 | MGMT 33 | 114 | 5 | Assay Validation | Negative | 0.02, 0.02 |
| 34 | MGMT 34 | 113 | 5 | Assay Validation | Negative | 0.0, 0.0 |
| 35 | MGMT 35 | 193 | 5 | Assay Validation | Negative | 0.02, 0.02 |
| 36 | MGMT 36 | 147 | 5 | Assay Validation | Negative | 0.0, 0.0 |
| 37 | MGMT 37 | 105 | 5 | Assay Validation | Negative | 0.0, 0.0 |
| 38 | MGMT 38 | 129 | 5 | Assay Validation | Negative | 0.0, 0.0 |
| 39 | MGMT 39 | 114 | 5 | Assay Validation | Negative | 0.0, 0.0 |
| 40 | MGMT 40 | 101 | 5 | Assay Validation | Negative | 0.14, 0.14 |

*information not provided

**Supplemental Table V3:** Sensitivity Results

| **Sensitivity Set 1** | **PMR (%)** | **Sensitivity Set 2** | **PMR (%)** |
| --- | --- | --- | --- |
| MGMT-1 | 103.4 | MGMT-10 | 15.4 |
| MGMT-2 | 128.4 | MGMT-11* | 0.9 |
| MGMT-3 | 27.5 | MGMT-12 | 3.3 |
| MGMT-4 | 8.1 | MGMT-13 | 30.3 |
| MGMT-5 | 112.4 | MGMT-14 | 53.1 |
| MGMT-6 | 8.4 | MGMT-15 | 47.2 |
| MGMT-7 | 12.9 | MGMT-16 | 141.3 |
| MGMT-8 | 7.9 |  |  |
| Positive control | 100 | Positive control | 100 |
| Negative control | 0.0 | Negative control | 0.0 |

**Supplemental Table V4:** Specificity assessment

| **Sensitivity Set 1** | **PMR (%)** | **Sensitivity Set 2** | **PMR (%)** |
| --- | --- | --- | --- |
| MGMT-21 | 0.0 | MGMT-32 | 0.1 |
| MGMT-23 | 0.0 | MGMT-33 | 0.9 |
| MGMT-24 | 0.1 | MGMT-34 | 0.0 |
| MGMT-25 | 0.0 | MGMT-37 | 0.0 |
| MGMT-26 | 0.0 | MGMT-38 | 0.0 |
| MGMT-27 | 0.1 | MGMT-39 | 0.0 |
| MGMT-29 | 0.0 | MGMT-40 | 0.5 |
| MGMT-30 | 0.0 |  |  |
| Positive control | 100.00 | Positive control | 100.00 |
| Negative control | 0.0 | Negative control | 0.0 |

**Supplementary Table V5:** Reproducibility Results

| **Sample** | **Operator 1 Rep 1 (%)** | **Operator 1 Rep 2 (%)** | **Operator 2 Rep 1 (%)** | | **Operator 2 Rep 2 (%)** |
| --- | --- | --- | --- | --- | --- |
| MGMT-9 | 137.4 | 116.0 | | 130.2 | 126.2 |
| MGMT-13 | 8.7 | 6.4 | | 9.5 | 11.2 |
| MGMT-14 | 128.9 | 123.2 | | 149.7 | 120.9 |
| MGMT-19 | 151.0 | 117.3 | | 156.1 | 135.3 |
| MGMT-20 | 21.3 | 18.1 | | 21.2 | 24.4 |
| MGMT-22 | 0.0 | 0.0 | | 0.0 | 0.0 |
| MGMT-28 | 0.0 | 0.0 | | 0.0 | 0.0 |
| MGMT-31 | 0.0 | 0.0 | | 0.0 | 0.0 |
| MGMT-35 | 0.0 | 0.0 | | 0.0 | 0.0 |
| MGMT-36 | 0.0 | 0.0 | | 0.0 | 0.0 |
| Positive control | 100.0 | 100.0 | | 100.0 | 100.0 |
| Negative control | 0.0 | 0.0 | | 0.0 | 0.0 |

# Supplementary Figures

**Supplementary Figure 1: Genes associated with response or resistance to veliparib plus temozolomide.**


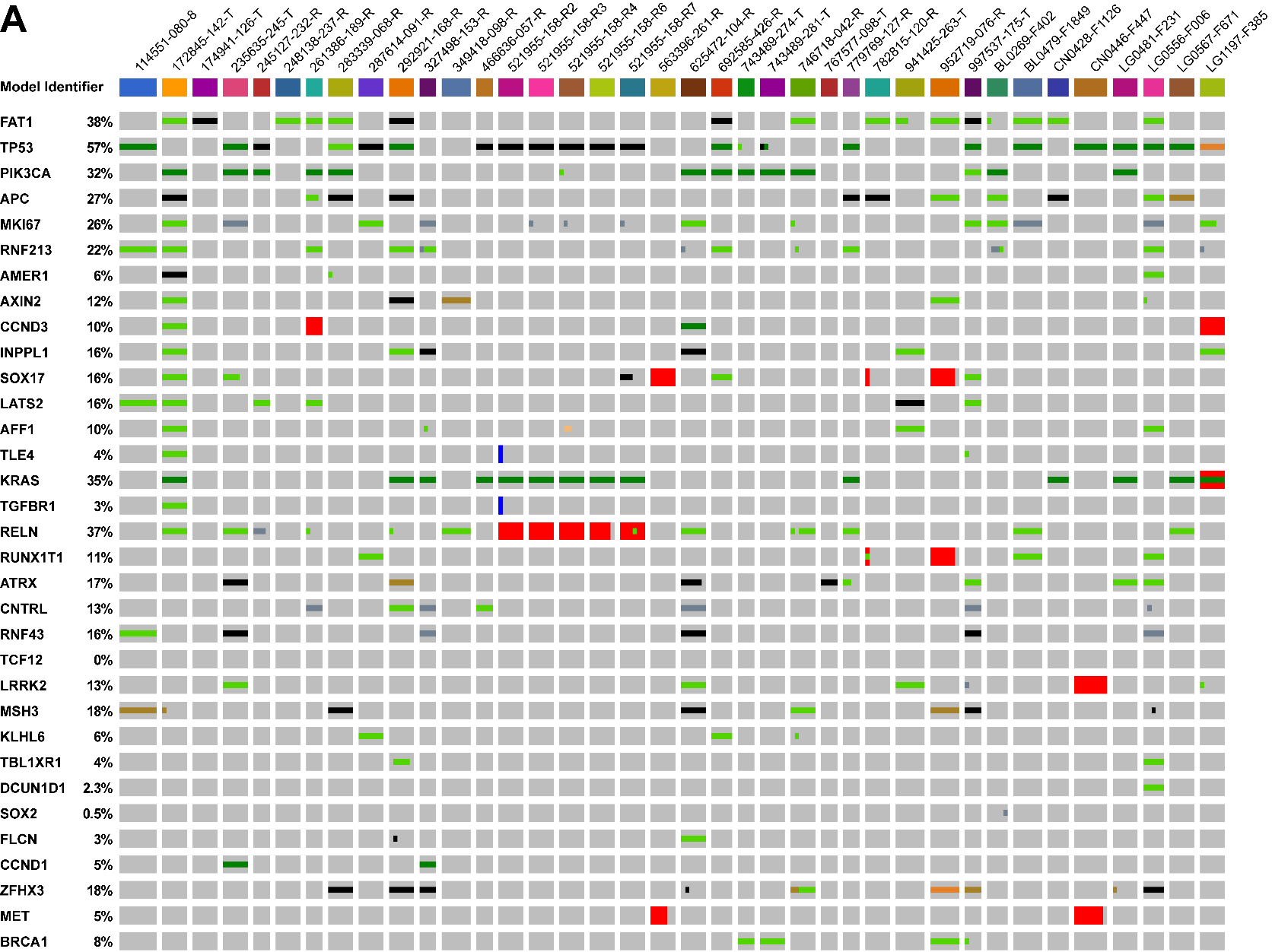


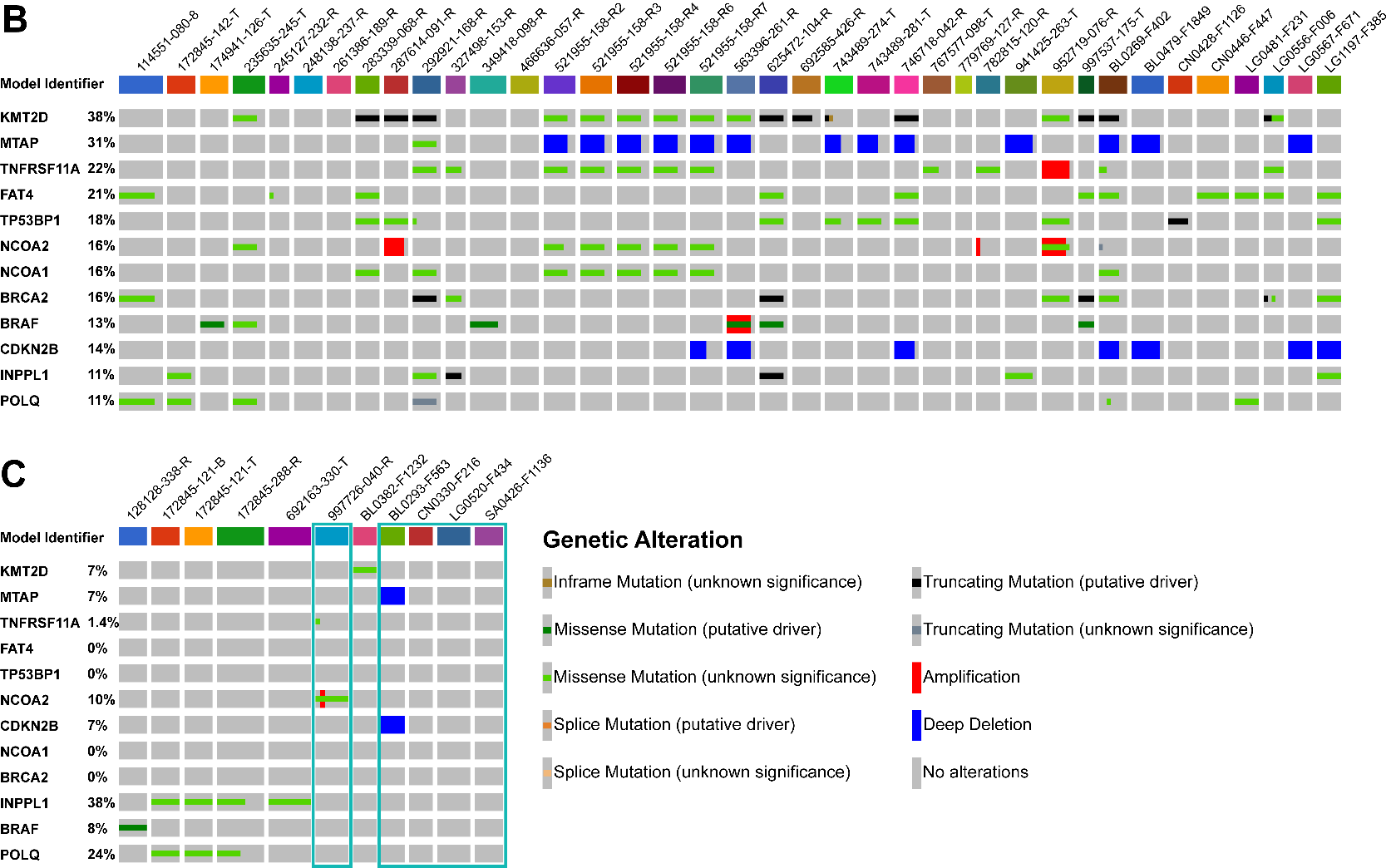


A) OncoPrint map illustrating the status of the most prevalent genetic alterations associated with response to veliparib plus temozolomide (see Figure 2), as detected in models resistant to the combination therapy. A minimum of 3 samples were analyzed per model; column width is determined by the number of samples analyzed. Percentages of samples harboring a genetic alteration are presented next to the gene symbol. B) OncoPrint map illustrating the most prevalent genetic alterations in the PDX models resistant to veliparib plus temozolomide. A minimum of 3 samples were analyzed per model; column width is determined by the number of samples analyzed. Percentages of samples harboring a genetic alteration are presented next to the gene symbol. C) OncoPrint map illustrating the most prevalent genetic alterations in the PDX models resistant to veliparib plus temozolomide as detected in the 11 PDX models that responded to the combination treatment. Column width is determined by the number of samples analyzed

**Supplementary Figure 2: Mismatch DNA repair genes in the context of *MGMT* deficiency.**


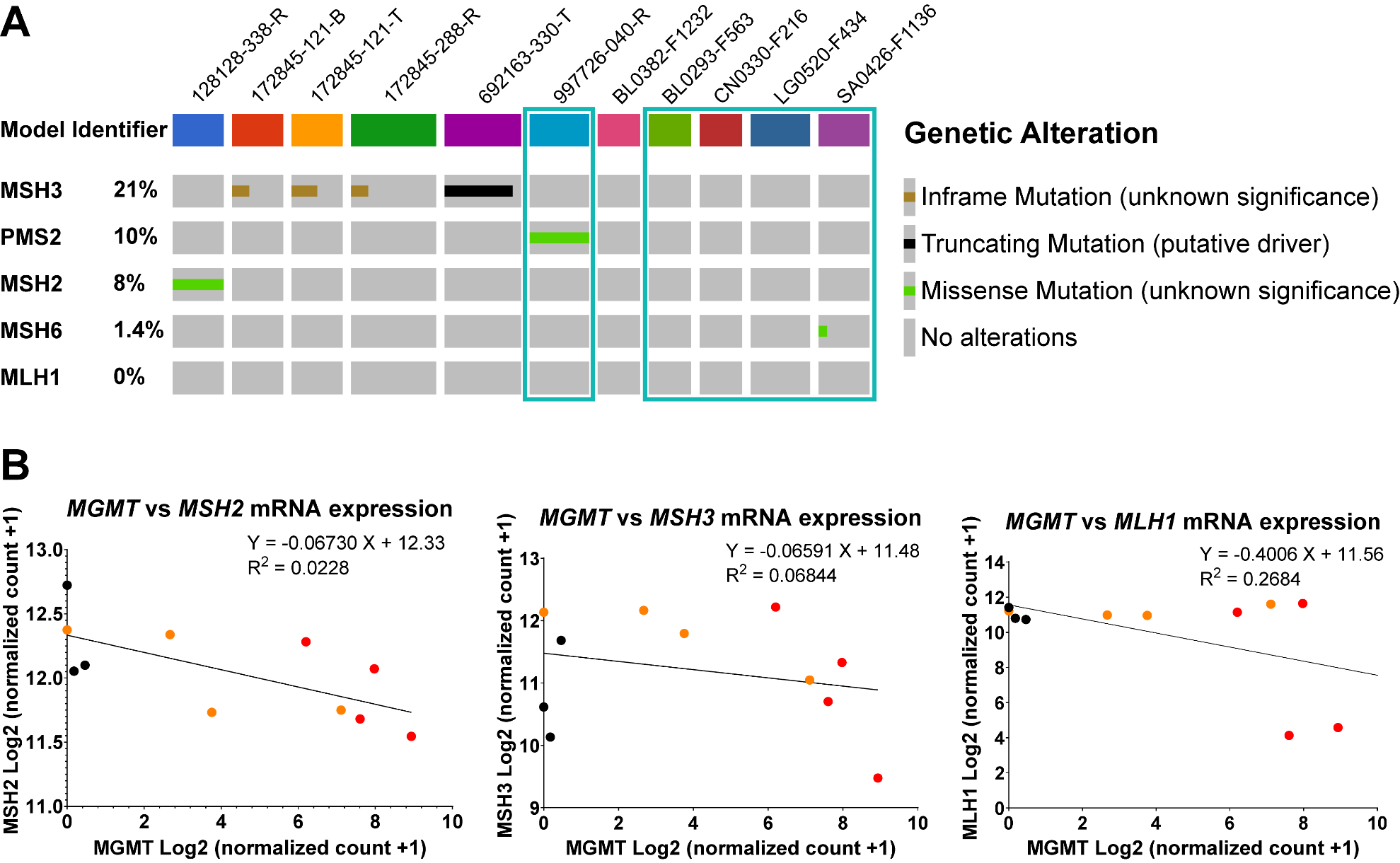


A) OncoPrint map showing genomic alterations of the DNA mismatch repair (MMR) pathway in the 11 PDX models that responded to veliparib plus temozolomide. Percentages of samples harboring a genetic alteration are presented next to the gene symbol. Highlighted in teal: models responding to temozolomide (Tmz) single treatment. B) Correlation between *MGMT* mRNA levels and MMR genes mRNA levels. Only samples with methylated *MGMT* promoters are represented. Averaged log2[normalizedCount+1] values represented for each model. In black: PDX models that responded to both single agent temozolomide and veliparib plus temozolomide treatment; in orange: PDX models that responded to veliparib plus temozolomide treatment but not single agent treatment; in red: PDX models that didn’t respond to either temozolomide single agent or combination treatment. Simple linear regression calculated using GraphPad Prism 10.1.

**Supplementary Figure 3: Median tumor volume showing PDX tumor responses to temozolomide-based therapy depending on *MGMT* promoter methylation status.**


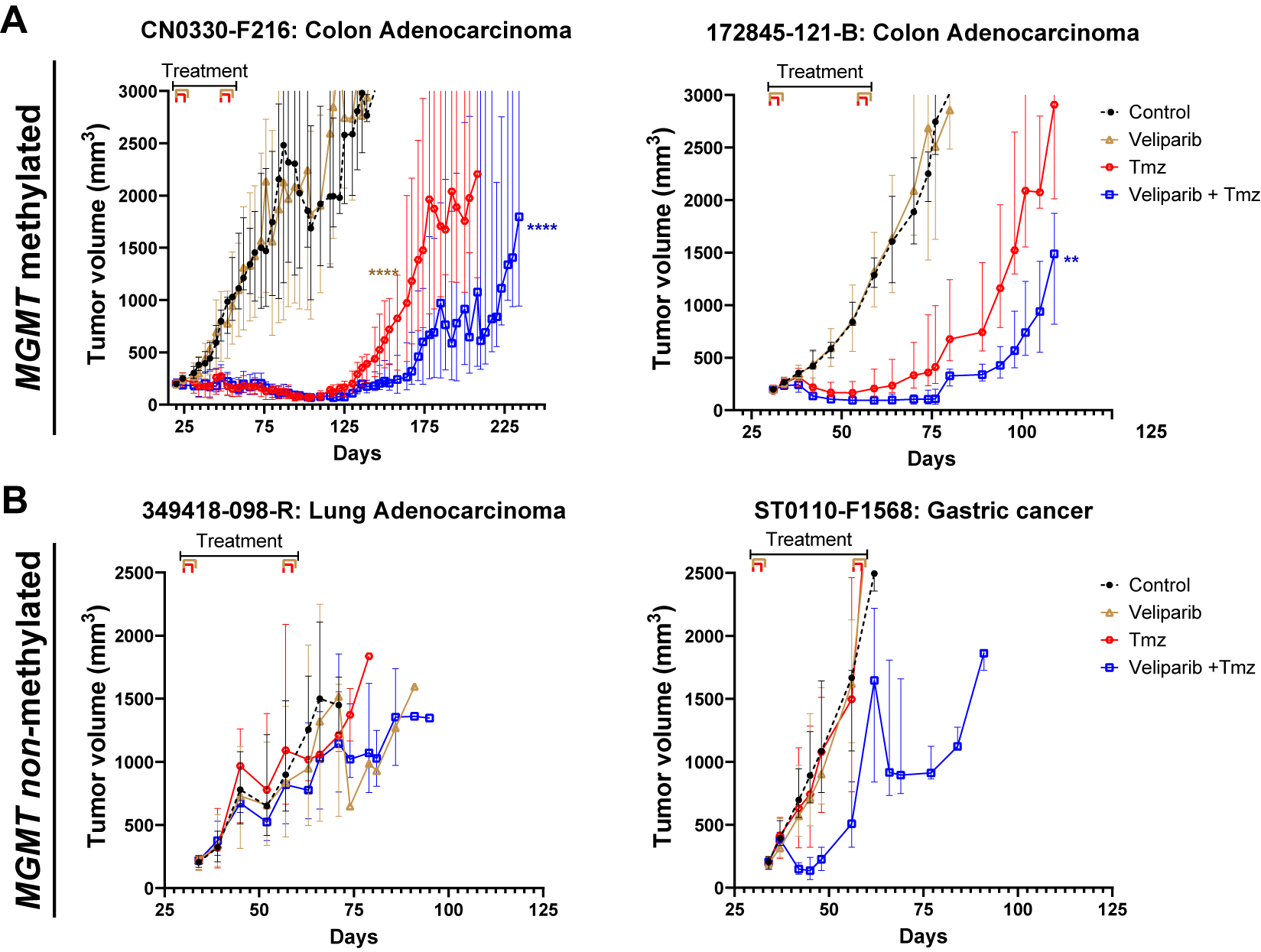


A) PDX models with methylated *MGMT* promoters that responded to temozolomide-based therapy. Left: CN00330-F216 colon adenocarcinoma model (temozolomide EFS x 4 = 3.1, PR; veliparib plus temozolomide EFS x 4 = 3.7, PR). Right: 172845-121-B colon adenocarcinoma model (temozolomide EFS x 4 = 1.7; veliparib plus temozolomide EFS x 4 = 2, PR. B) PDX models with non-methylated *MGMT* promoters that did not respond to temozolomide-based therapy. Left: 349418-098-R lung adenocarcinoma model (temozolomide EFS x 4 = 0.9; veliparib plus temozolomide EFS x 4 = 1.2). Right: ST0110-F1568 gastric cancer model (temozolomide EFS x 4 = 1.1; veliparib plus temozolomide EFS x 4 = 1.3). Error bars: 95% confidence interval. Treatment duration indicated on top. (** *p* < 0.01; **** *p* < 0.0001; Kruskal Wallis analysis and Dunn’s multiple comparison test).

**Supplementary Figure 4: MGMT expression in PDX models.**


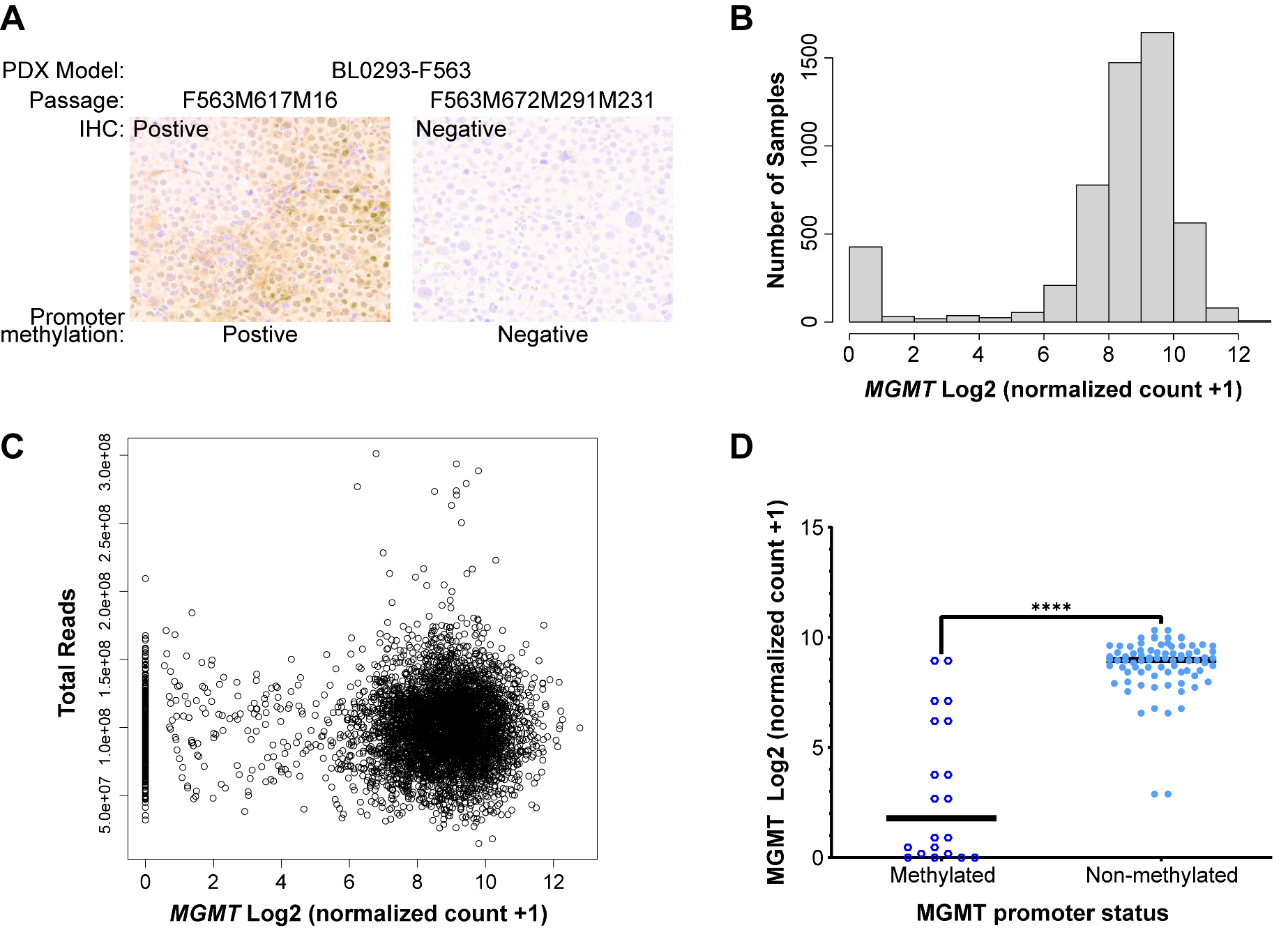


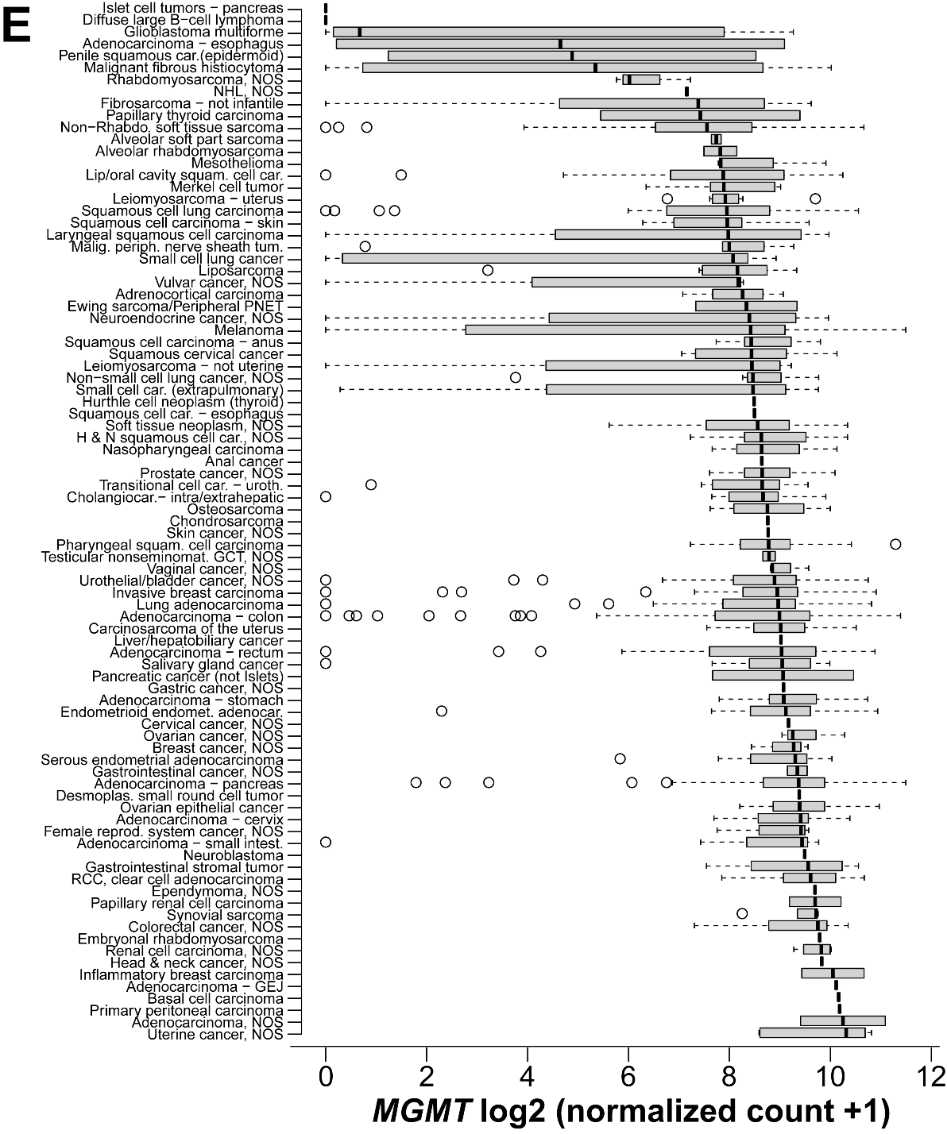


A) MGMT protein expression as detected by immunohistochemistry (IHC) staining in model BL0293-F563, the only tested model for which MGMT protein expression changed from positive to negative across different passages. B) *MGMT* mRNA expression (RSEM [RNA-Seq by Expectation-Maximization] quantification) in 5348 PDMR specimens derived from 1056 PDX models. C) *MGMT* mRNA expression compared to total mRNA reads in 5348 PDMR specimens derived from 1056 PDX models. D) *MGMT* mRNA expression (RSEM [RNA-Seq by Expectation-Maximization] quantification) compared to *MGMT* promoter methylation status in preclinical MPACT models. Two samples were analyzed per model. Probability value (two tailed T-test) **** *p* < 0.0001. E) *MGMT* mRNA expression (RSEM [RNA-Seq by Expectation-Maximization] quantification) in 1056 PDMR PDX models according to cancer type. Median with 25%-75% quartile.

# Supplementary References

1. Li H, Durbin R. Fast and accurate short read alignment with Burrows-Wheeler transform. *Bioinformatics*. (2009) 25:1754-60. doi: 10.1093/bioinformatics/btp324

2. Kendig KI, Baheti S, Bockol MA, Drucker TM, Hart SN, Heldenbrand JR, et al. Sentieon DNASeq Variant Calling Workflow Demonstrates Strong Computational Performance and Accuracy. *Front Genet*. (2019) 10:736. doi: 10.3389/fgene.2019.00736

3. DePristo MA, Banks E, Poplin R, Garimella KV, Maguire JR, Hartl C, et al. A framework for variation discovery and genotyping using next-generation DNA sequencing data. *Nat Genet*. (2011) 43:491-8. doi: 10.1038/ng.806

4. Rimmer A, Phan H, Mathieson I, Iqbal Z, Twigg SRF, Consortium WGS, et al. Integrating mapping-, assembly- and haplotype-based approaches for calling variants in clinical sequencing applications. *Nat Genet*. (2014) 46:912-8. doi: 10.1038/ng.3036

5. Langmead B, Trapnell C, Pop M, Salzberg SL. Ultrafast and memory-efficient alignment of short DNA sequences to the human genome. *Genome Biol*. (2009) 10:R25. doi: 10.1186/gb-2009-10-3-r25

6. Li B, Dewey CN. RSEM: accurate transcript quantification from RNA-Seq data with or without a reference genome. *BMC Bioinformatics*. (2011) 12:323. doi: 10.1186/1471-2105-12-323

7. de Abreu FB, Gallagher TL, Liu EZ, Tsongalis GJ. Determining methylation status of methylguanine DNA methyl transferase (MGMT) from formalin-fixed, paraffin embedded tumor tissue. *MethodsX*. (2014) 1:42-8. doi: 10.1016/j.mex.2014.06.001

8. Tost J, Gut IG. DNA methylation analysis by pyrosequencing. *Nat Protoc*. (2007) 2:2265-75. doi: 10.1038/nprot.2007.314
